# The impact of postpartum obsessive-compulsive symptoms on child development and the mediating role of the parent–child relationship: A prospective longitudinal study

**DOI:** 10.3389/fpsyt.2022.886347

**Published:** 2022-08-16

**Authors:** Sophie Blum, Judith T. Mack, Victoria Weise, Marie Kopp, Eva Asselmann, Julia Martini, Susan Garthus-Niegel

**Affiliations:** ^1^Institute and Outpatient Clinics of Occupational and Social Medicine, Faculty of Medicine, Technische Universität Dresden, Dresden, Germany; ^2^Department of Psychology, HMU Health and Medical University, Potsdam, Germany; ^3^Institute of Clinical Psychology and Psychotherapy, Technische Universität Dresden, Dresden, Germany; ^4^Department of Psychiatry and Psychotherapy, Faculty of Medicine, Carl Gustav Carus University Hospital, Technische Universität Dresden, Dresden, Germany; ^5^Institute for Systems Medicine and Faculty of Medicine, Medical School Hamburg, Hamburg, Germany; ^6^Department of Child Health and Development, Norwegian Institute of Public Health, Oslo, Norway

**Keywords:** postpartum obsessive-compulsive symptoms, mental health, child development, mother–child relationship, father–child relationship, bonding, DREAM study

## Abstract

**Background:**

The first 2 years of life are a particularly sensitive period for the parent–child relationship as well as a healthy, age-appropriate child development. Both have been shown to be linked to postpartum depressive and anxiety symptoms, while the role of obsessive-compulsive symptoms, which are also common, is still largely understudied. In addition, fathers have been neglected in this area of research. This study, which includes both mothers and fathers, aims to investigate the longitudinal associations between postpartum obsessive-compulsive symptoms and different domains of child development, as well as the mediating role of the parent–child relationship.

**Methods:**

Data were drawn from the prospective longitudinal study DREAM, with 674 mothers and 442 fathers from the general population completing self-report questionnaires at four measurement points. Longitudinal associations between parental postpartum obsessive-compulsive symptoms 8 weeks postpartum, the parent–child relationship 14 months postpartum, and child development 24 months postpartum were investigated using regression and mediation analyses. A number of potential confounding variables were considered, i.e., age, academic degree, postpartum depressive and anxiety symptoms of the parents, preterm birth and temperament of the child, as well as COVID-19 pandemic-driven adversities.

**Results:**

When adjusting for confounders, neither maternal nor paternal postpartum obsessive-compulsive symptoms had adverse effects on the respective parent–child relationship and child development. Further, no mediating role of the parent–child relationship between parental postpartum obsessive-compulsive symptoms and child development could be confirmed. Instead, we found that the mother– and father–child relationship were differentially related to specific child developmental domains. For mothers, a poorer mother–child relationship was prospectively related to poorer fine motor development. For fathers, a poorer father–child relationship prospectively predicted a poorer overall development as well as poorer gross motor, fine motor, problem-solving, and personal-social development.

**Conclusion:**

Our results suggest that negative effects on the parent–child relationship and child development may only become apparent in full-blown postpartum obsessive-compulsive disorder. Given the differential impact on specific developmental domains, our findings also suggest that it is crucial to consider both parents in clinical practice as well as in future research, rather than focusing only on the mother–child dyad.

## Introduction

Obsessive-compulsive disorder is a common, often chronic mental health condition with an estimated lifetime prevalence between 1 and 2% in the general population (1–3). In addition, 13–28% experience subthreshold obsessive-compulsive symptoms throughout their life (3, 4). Obsessive-compulsive disorder is characterized by recurrent intrusive thoughts, images or impulses (obsessions), and/or repetitive behavioral/mental acts (compulsions). Affected individuals suffer from unwanted obsessions that are ego-dystonic, i.e., not in line with their personal values and intentions, and cause significant anxiety or emotional distress, often resulting in time-consuming, debilitating compulsions to reduce unpleasant feelings or avoid potential harm (5).

In the existing literature, it is well documented that obsessive-compulsive disorder usually not only has a major impact on the psychological well-being, psychosocial functioning, and quality of life of affected individuals (6–10), but often also leads to considerable family burden and reduced quality of life among non-affected relatives (11–13). Furthermore, the results of a study in a clinical sample by Black and colleagues revealed that 7–18-year-old children of parents with obsessive-compulsive disorder are significantly more likely to be affected by social, behavioral, and emotional disorders including obsessive-compulsive disorder, compared to children from a healthy control group (14). Moreover, recent population-based studies indicate that even the presence of subthreshold maternal obsessive-compulsive symptoms is associated with an increased risk of developing obsessive-compulsive symptoms (15, 16) as well as general emotional and behavioral problems in their school-aged children (16).

However, despite the above-mentioned knowledge concerning the detrimental impact of obsessive-compulsive symptoms on close family members, to date there are no empirical studies addressing the potential adverse effects of parental obsessive-compulsive symptoms on the well-being and development of younger children, i.e., their infants and/or toddlers. This is especially problematic because in the first years of life children are highly dependent on their parents and their affectionate caregiving. In addition, neuroscientific studies indicate that the first 2 years of life are a particularly sensitive period for a child’s healthy and age-appropriate development, characterized by rapid brain growth and highly dynamic brain development (17–20). Accordingly, adverse social experiences in early childhood are associated with altered brain volume, structure, and functioning (21–24) persisting at least into young adulthood (23). At the same time, there is evidence that the first 2 years of life are also a period in which psychosocial interventions are most effective in preventing later developmental delays in children (25, 26). Taken together, these findings highlight the need and urgency to identify at-risk infants and toddlers as early as possible in order to provide them and their parents with adequate interventions and avoid or at least mitigate harmful long-term consequences.

For expectant parents, childbirth and the transition to parenthood may represent stressful life events characterized by new responsibilities for a little human being and a variety of profound life changes and challenges, usually affecting the entire family system. As a result, the postpartum period is recognized as a time of heightened vulnerability to the development or exacerbation of mental health issues in new mothers and fathers (27, 28), often entailing both considerable personal and socio-economic costs (29).

In this context, a large number of studies demonstrated that poor mental health in the postpartum period is often associated with various negative outcomes for both parents and their offspring. For instance, maternal postpartum depressive symptoms are frequently linked to poorer health-related quality of life (e.g., 30, 31), greater partnership difficulties (e.g., 32, 33), increased suicidality (e.g., 34, 35), and poorer quality of mother–child relationship (e.g., 36–41). Even though less well established in comparison to maternal postpartum depressive symptoms, similar results have been found with respect to paternal postpartum depressive symptoms (28, 42). Furthermore, numerous studies have shown that postpartum depressive symptoms are often associated with poorer early child development in various domains, such as achieving age-appropriate cognitive (e.g., 43, 44), motor (e.g., 43, 45), language (e.g., 46, 47), and social-emotional (e.g., 48) milestones (for recent systematic reviews, see 49, 50).

In comparison to the extensive literature on postpartum depressive symptoms, research investigating the relationship between postpartum anxiety symptoms and their consequences for the offspring is still quite scarce, partly ambiguous, and suffers from a number of methodological limitations (e.g., small sample sizes, cross-sectional designs, and/or no investigation of fathers). Nevertheless, several prior studies also provide growing evidence that higher levels of postpartum anxiety symptoms are most frequently related to a deterioration in early mother–child relationship (36, 38, 40, 51–55), although in some studies this association was no longer significant after adjusting for postpartum depressive symptoms (36, 55) and in one study postpartum anxiety symptoms were even inversely associated with mother–child relationship and thus had a positive influence on bonding quality compared to depressed and healthy mothers (56). Concerning child development, the few existing studies revealed that postpartum anxiety symptoms seem most likely to be related to both poorer social-emotional development (57–59) as well as poorer language development (60) in young children. However, other studies failed to show any direct impact on cognitive (60–62), motor (57, 62), or language development (57, 62) in early childhood. Nevertheless, even if postpartum anxiety symptoms *per se* were not consistently related to poorer child development, one risk factor was found that significantly predicted poorer developmental trajectories in offspring from highly anxious parents, namely, parental avoidance behavior (60).

A symptomatology that remains often overlooked in the postpartum period (63, 64) and that is usually accompanied by marked avoidance behavior (65–68), are postpartum obsessive-compulsive symptoms. Experiencing postpartum obsessive-compulsive symptoms is a highly prevalent phenomenon among both new mothers and fathers, with the vast majority of new parents reporting transient intrusive thoughts (69–73). Moreover, based on a recent large-scale longitudinal study from Canada, the weighted period prevalence for obsessive-compulsive disorder (DSM-5) in the first 9 months postpartum is estimated to be 16.9%, with a point prevalence peak of 8.7% at 8 weeks after delivery (74). In addition, the content of obsessions and compulsions in the postpartum period is often infant-related. Hence, the parents’ intrusive and ego-dystonic thoughts, images, or impulses often focus on the child’s health and well-being, with aggressive obsessions of accidental and/or intentional infant-related harm being the most common (68, 70, 75–77). Nevertheless, despite the specific clinical picture and high prevalence rate, the potential adverse impact of postpartum obsessive-compulsive symptoms on parent–child relationship and child development remains widely understudied compared to that of postpartum depressive and anxiety symptoms.

**FIGURE 1:**
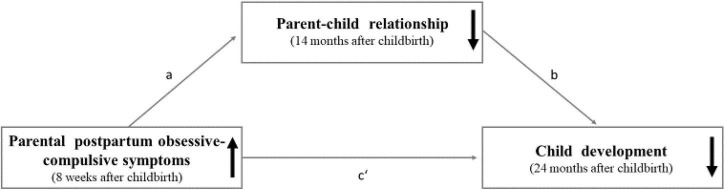
The proposed mediation model with the hypothesized prospective longitudinal associations between parental postpartum obsessive-compulsive symptoms (predictor), parent-child relationship (mediator), and child development (outcome).

Given these knowledge gaps, the aim of the present study is to investigate the prospective longitudinal associations between postpartum obsessive-compulsive symptoms in mothers and fathers 8 weeks after childbirth, parent–child relationship 14 months after childbirth, and child development 24 months after childbirth. Additionally, we will explore whether the associations between postpartum obsessive-compulsive symptoms and child development are mediated by the parent–child relationship (see Figure 1).

In this context, we propose the following four hypotheses: First, we predict that higher levels of parental postpartum obsessive-compulsive symptoms will be associated with poorer child development. Second, we hypothesize that higher levels of postpartum obsessive-compulsive symptoms will be related to a poorer parent–child relationship. Third, we predict that a poorer parent–child relationship will be associated with poorer child development. Fourth, we hypothesize that the parent–child relationship will mediate the association between parental postpartum obsessive-compulsive symptoms and child development. Further, a number of potential confounding variables (age, academic degree, postpartum depressive and anxiety symptoms of the parents, preterm birth, temperament of the child, and coronavirus disease 2019 (COVID-19) pandemic-driven adversities) will be considered. The obtained results will contribute to the knowledge concerning the etiology of developmental delays in young children and could provide an evidence base for tailored prevention and early intervention approaches helping to promote the child’s well-being and healthy, age-appropriate development.

## Materials and methods

### Study design

The present investigation is part of the Dresden Study on Parenting, Work, and Mental Health (**DR**esdner Studie zu **E**lternschaft, **A**rbeit und **M**entaler Gesundheit, **DREAM**), a large population-based cohort study with a multi-method approach involving *n* = 3,863 individuals. The overall aim of the DREAM study is to prospectively investigate the complex interplay of factors related to parental working conditions, role distribution, and psychological stress and their potential impact on peripartum outcomes as well as on long-term physical and mental health in parents and their offspring from pregnancy to middle childhood. Currently, the study consists of six measurement points: during pregnancy (T1), 8 weeks after the anticipated birth date (T2), 14 months (T3), 2 years (T4), 3 years (T5), and 4.5 years (T6) after the actual birth date. Between June 2017 and end of 2020, expectant mothers and their male or female partners were mainly recruited at obstetrical clinics and midwife practices in and around the city of Dresden, Germany. Criteria for inclusion were becoming parent of a newborn child during the aforementioned recruitment period, residency in Dresden or the surrounding area, and sufficient German language skills to answer the questionnaires. Approached (expectant) parents had the choice to participate in the study either alone or together as a couple. More detailed information concerning the rationale, design, and methods of the DREAM study are provided in the study protocol (78).

### Sample

**FIGURE 2:**
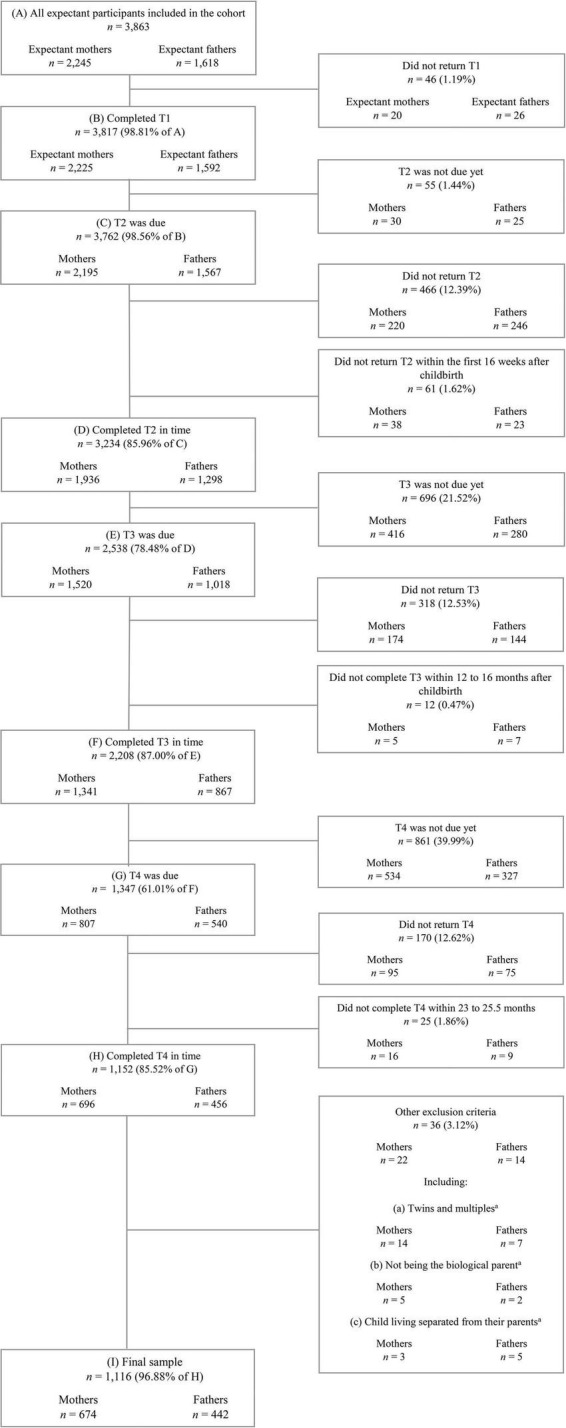
Flowchart of the retention process and exclusion criteria resulting in the final sample. T1, during pregnancy; T2, 8 weeks after the anticipated birth date; T3, 14 months after the actual birth date; T4, 24 months after the actual birth date. Data from March 29th, 2021 (prospective data collection ongoing). *^a^*Exclusion criteria met or missing data in these variables.

In this study, we analyzed data from participating mothers and fathers who had completed the first four measurement points within a previously defined time frame until the day of data extraction (March 29th, 2021). The detailed retention process and exclusion criteria resulting in the current sample are presented in a flowchart in Figure 2. Due to the developmental sensitivity of the studied constructs, timely completion of the questionnaires was defined for each measurement point individually. Accordingly, in this study we excluded all participants who did not complete T1 at all, completed T2 not within the first 16 weeks postpartum, T3 not within 12–16 months postpartum, or T4 not within 23–25.5 months postpartum. Furthermore, we excluded parents of twins or multiples, non-biological parents, children living separated from their parents, and parents with missing data in these variables. By applying these criteria, the final sample included 1,116 individuals, consisting of *n* = 674 mothers and *n* = 442 fathers.

### Measures

#### Parental postpartum obsessive-compulsive symptoms

Parental postpartum obsessive-compulsive symptoms were measured at T2 with a subscale of the validated German version of the Symptom-Checklist Revised (SCL-90-R; 79). The SCL-90-R is a widely used multidimensional self-report screening instrument designed to assess a broad range of psychological symptoms and distress covering nine different symptom dimensions, with one of them being obsessive-compulsive (80). The obsessive-compulsive subscale comprises 10 items asking the person completing the questionnaire to indicate their psychological burden in the past 7 days on a five-point Likert scale ranging from *not at all* (0) to *extremely* (4). Individuals with high scores on the obsessive-compulsive subscale suffer from recurrent intrusive thoughts which they cannot get rid of, memory, decision-making, and concentration problems, worrying about being careless, difficulties to start something, and/or compulsive behavior, e.g., repetitive checking, counting, or washing (79). A total score was calculated by summing up the item scores, resulting in a range between 0 and 40. Higher total scores reflect higher levels of obsessive-compulsive symptom burden. In the current study, the reliability of the obsessive-compulsive subscale at T2 was very good [Cronbach’s α = 0.83 (mothers) and α = 0.83 (fathers)].

#### Parent–child relationship

Parent–child relationship was measured at T3 with the validated German version of the Postpartum Bonding Questionnaire (PBQ; 81, 82). The PBQ is a widely used self-report screening instrument designed to identify risks for disorders in the parent–child relationship in the postpartum period (83). It comprises 25 items and rates the parent–child relationship in four domains: impaired bonding, rejection and anger, anxiety about care, and risk of abuse. Parents are asked to think of the most difficult time with their child and are asked to answer each item on a six-point Likert scale ranging from *never* (0) to *always* (5). A total score was calculated by summing up the item scores of each subscale, resulting in a range between 0 and 125. Higher total scores reflect a poorer parent–child relationship. In the current study, the reliability of the PBQ at T3 was very good to excellent [Cronbach’s α = 0.91 (mothers) and α = 0.87 (fathers)].

#### Child development

Child development was measured at T4 with a German version of the 24-months version of the Ages and Stages Questionnaire-3 (ASQ-3; 84). First, we translated the ASQ-3 from English into German. Second, a native speaker back-translated the German version into English. Finally, we adapted items of the German version where needed. The ASQ-3 is an internationally used parent-rated screening instrument designed to identify risks for developmental delays in young children. It comprises 30 items and rates the age-specific child development in five different domains: communication, gross motor, fine motor, problem-solving, and personal-social development. Depending on whether and how often the child demonstrates the developmental skills, caregivers are asked to answer each item as *yes* (0), *sometimes* (5), or *not yet* (10). For each domain, a domain-specific score was calculated by summing up the item scores, resulting in a range between 0 and 60. Moreover, a total score was calculated by summing up the five domain-specific scores, resulting in a range between 0 and 300. In accordance with Squires et al. (84), we recoded the items so that higher scores reflect a better child development. In the current study, the reliability of the ASQ-3 total score at T4 was good to very good [Cronbach’s α = 0.76 (mothers) and α = 0.82 (fathers)]. However, the reliability of the domain-specific scores was rather moderate to poor [mothers: α = 0.70 (communication); α = 0.60 (gross motor); α = 0.40 (fine motor); α = 0.47 (problem-solving); α = 0.32 (personal-social), and fathers: α = 0.67 (communication); α = 0.57 (gross motor); α = 0.45 (fine motor); α = 0.52 (problem-solving); α = 0.47 (personal-social)].

#### Potential confounding variables

Based on prior research findings, it was assumed that the following six parent- and child-related factors may be significantly associated with the mediator (parent–child relationship) and outcome (child development) variables: age, academic degree, postpartum depressive and anxiety symptoms of the parents, as well as preterm birth and temperament of the child. For this reason, in accordance with the recommendations of Lee (85), these factors were considered as theoretical confounding variables in all analyses regardless of their empirical evidence in the present study. In addition, COVID-19 pandemic-driven adversities were incorporated as another potential confounding variable given the preliminary evidence suggesting the restrictions in the context of pandemics (e.g., social distancing and shutdowns) may be a risk factor for the onset and/or exacerbation of unfavorable environmental circumstances, which in turn may represent a risk factor for developmental delays in children (86).

Parental age and academic degree were both measured at T1. Parental age was assessed in years. Parental academic degree was assessed with a question based on the German National Cohort Consortium (87) and was subsequently dichotomized as 1 (*completed university degree*) or 0 (*no completed university degree*).

Parental postpartum depressive and anxiety symptoms were both measured at T2. Parental postpartum depressive symptoms were assessed with the validated German version of the Edinburgh Postnatal Depression Scale (EPDS; 88, 89). The EPDS is the most common used self-report screening instrument designed to assess the frequency of depressive symptoms in the postpartum period (90). It comprises 10 items asking the person completing the questionnaire to indicate their psychological burden in the past 7 days on a four-point Likert scale. Individuals with high scores on the EPDS suffer from anhedonia, feelings of sadness and guilt, excessive worrying and crying, sleep disturbances, mental overload, and/or suicidal ideation. A total score was calculated by summing up the item scores, resulting in a range between 0 and 30. Higher total scores reflect higher levels of depressive symptom burden. In the current study, the reliability at T2 was good to very good [Cronbach’s α = 0.81 (mothers) and α = 0.77 (fathers)]. Parental postpartum anxiety symptoms were measured with a subscale of the German version of the Symptom-Checklist Revised (SCL-90-R; 79). The anxiety subscale comprises 10 items asking the person completing the questionnaire to indicate their psychological burden in the past 7 days on a five-point Likert scale ranging from *not at all* (0) to *extremely* (4). Individuals with high scores on the anxiety subscale suffer from nervousness, tremors, panic attacks, fearfulness, palpitations, tachycardia, agitation, the feeling that something bad is going to happen to them, and/or terrifying thoughts (79). A total score was calculated by summing up the item scores, resulting in a range between 0 and 40. Higher total scores reflect higher levels of anxiety symptom burden. In the current study, the reliability of the anxiety subscale at T2 was moderate to good [Cronbach’s α = 0.77 (mothers) and α = 0.65 (fathers)].

Preterm birth and child temperament were both measured at T2. Preterm birth was assessed by calculating the week of gestation and subsequently dichotomized as 1 (*yes, birth before the 37th week of gestation*) or 0 (*no, full-term birth*). Child temperament was assessed with a subscale of the German version of the Infants Characteristics Questionnaire (ICQ; 91). The ICQ is a widely used parent-rated screening instrument designed to assess the parents’ perception of the early infant temperament. The fussy/difficult subscale comprises nine items asking the caregivers to rate the infant’s behavior on a seven-point Likert scale. A total score was calculated by summing up the item scores, resulting in a range between 0 and 63. Higher total scores reflect a more difficult and fussy temperament. In the current study, the reliability of the fussy/difficult subscale at T2 was very good [Cronbach’s α = 0.86 (mothers) and α = 0.86 (fathers)].

COVID-19 pandemic-driven adversities were accounted for with the date of completion of T4, i.e., the date of the parent-reported scores of child development. Date of completion was dichotomized into 0 (*before the first COVID-19 pandemic-driven adversities in Germany, i.e., between June 2019 and March 2020*) vs. 1 (*after the first COVID-19 pandemic-driven adversities, i.e., between March 2020 and the time of data extraction in March 2021*).

### Statistical analyses

All statistical analyses were performed using IBM SPSS Statistics (Version 27) and the SPSS modeling tool PROCESS (92). For mothers and fathers, all analyses were performed separately. The alpha level was set at 0.05. Due to missing data of some participants, the number of observations varied slightly between the analyses. In a first step, sum scores and Cronbach’s α for all psychometric scales were calculated. Missing items of the SCL-R, EPDS, PBQ, and ICQ were replaced by the individual’s mean, if no more than 20% of the items were missing. For the ASQ-3, following the recommendations of Squires et al. (84), missing items of the subscales were replaced by the participant’s mean of the respective subscale, as long as no more than two items were missing. In addition, we ensured that at least 80% of all items were answered before calculating the ASQ-3 total score. Subsequently, descriptive analyses were conducted for all sociodemographic characteristics of the sample as well as for the predictor, mediator, outcome, and potential confounding variables. Furthermore, bivariate analyses were performed to determine the direction and strength of the respective associations among all predictor, mediator, outcome, and potential confounding variables. For this purpose, Pearson’s correlation coefficients were calculated. Next, dropout analyses were performed to explore whether participating individuals who completed all four measurement points differed with respect to sociodemographic characteristics, predictor, mediator, outcome, and/or potential confounding variables from those who did not complete T4.

The main analyses included linear regression and mediation analyses. Simple and multiple linear regression analyses were performed to investigate the prospective longitudinal associations between parental postpartum obsessive-compulsive symptoms, the parent–child relationship, and child development. Mediation analyses using model 4 of the SPSS modeling tool PROCESS (92) were conducted to investigate the hypothesized mediating role of the parent–child relationship. In this context, all aforementioned potential confounding variables were considered in the analyses by adding them to the models in a second step *via* forced entry.

For the mediation analyses, we calculated the total effect (*c*), the direct effect (*c*′), and the indirect effect (ab) based on ordinary least square regressions. However, in determining possible mediation effects, only the direct and indirect effects were interpreted, as recommended by Zhao et al. (93) and Rucker et al. (94). Indirect effects were considered as statistically significant if the 95% confidence interval (CI) did not cross zero.

Before running the regression and mediation analyses, the data sets of participating mothers and fathers were checked for implausible extreme values and influential outliers. All identified univariate extreme values, i.e., values deviating ±3 SD from the respective mean, were considered as contextually plausible and therefore included in all statistical analyses. Multivariate extreme values, i.e., cases deviating ±3 SD in the outcome variables, were identified using casewise diagnosis and standardized residuals. Their impact on the regression estimates was examined by calculating Cook’s distances (cut-off: <1; 95), leverage values (cut-off: <0.2; 96), and DFBETAS (cut-off: <|2|; 97). Since no influential cases were identified, all multivariate extreme values were included in the analyses. In addition to checking the data sets for extreme values and influential outliers, the main assumptions for linear models, i.e., linearity, homoscedasticity, independent standard errors, normally distributed standard errors, and no perfect multicollinearity, were tested by following the recommendations of Field (98). The aforementioned assumptions were partially confirmed. Due to violations regarding homoscedasticity and normally distributed standard errors, bootstrapping with 5,000 iterations was used to compute confidence intervals (CI) and inferential statistics for both the regression and mediation analyses to increase the confidence of the yielded results (98).

### Ethics statement

The Ethics Committee of the Medical Faculty of the Technische Universität Dresden approved all parts of the DREAM study (EK 278062015). Participants obtained written information concerning the key research goals and the study procedure prior to the first assessment. They were informed about pseudonymisation of the data and their right to withdraw from the study at any time. All participants signed an informed consent form.

## Results

### Sample characteristics

Overall, *n* = 674 mothers and *n* = 442 fathers were included in the final sample. Sample characteristics are shown in Table 1. During pregnancy, the age of expectant mothers and fathers ranged from 20–43 years (*M* = 30.12; *SD* = 3.84) and 22–56 years (*M* = 32.25; *SD* = 4.93), respectively. At this time, the majority of participating women and men were living in a committed relationship (mothers: 99.6%; fathers: 99.8%) and expecting their first child (mothers: 80.4%; fathers: 81.3%). In the present sample, most participants were born in Germany (mothers: 96.6%; fathers: 97.5%) and more than half of them had a university degree (mothers: 61.5%; fathers: 59.4%), indicating a higher academic degree level compared to the general population in Dresden (26.3%; 99). For both parents, mean scores for postpartum obsessive-compulsive symptoms (SCL-90-R; mothers: *M* = 4.33; *SD* = 4.10; fathers: *M* = 3.13; *SD* = 3.61), postpartum depressive symptoms (EPDS; mothers: *M* = 5.61; *SD* = 3.77; fathers: *M* = 3.44; *SD* = 3.10), and postpartum anxiety symptoms (SCL-90-R, mothers: *M* = 1.75; *SD* = 2.64; fathers: *M* = 1.28; *SD* = 1.89) were relatively low at T2, indicating rather low mental health symptom burden in the early postpartum period. Furthermore, most participants reported a good parent–child relationship quality at T3 (PBQ total score; mothers: *M* = 14.23; *SD* = 10.40; fathers: *M* = 13.43; *SD* = 8.33) and a good overall developmental status of their child at T4 (ASQ-3 total score; mothers: *M* = 260.96; *SD* = 26.93; fathers: *M* = 256.66; *SD* = 30.83). Most children were born at full-term (i.e., ≥ the 37th week of gestation; mothers: 96.3%; fathers: 96.8%) and were perceived by their parents as moderately fussy and difficult (ICQ; mothers: *M* = 28.79; *SD* = 7.93; fathers: *M* = 28.77; *SD* = 7.74).

**TABLE 1:** Sample description.

	Total sample (*n*^a^ = 1,116)			
	Mothers (*n*^a^ = 674)		Fathers (*n*^a^ = 442)	
	*M (SD)*	Range	*M (SD)*	Range
**Parental age in years** (T1)	30.12 (3.84)	20–43	32.25 (4.93)	22–56
**Week of (partner’s) pregnancy** (T1)	30.60 (5.81)	11–41	30.90 (5.93)	12–41
**Parental postpartum obsessive-compulsive symptoms** ^c^ (T2; 0–40)	4.33 (4.10)	0–26	3.13 (3.61)	0–21
**Parental postpartum depressive symptoms** ^d^ (T2; 0–30)	5.61 (3.77)	0–23	3.44 (3.10)	0–17
**Parental postpartum anxiety symptoms** ^e^ (T2; 0–40)	1.75 (2.64)	0–21	1.28 (1.89)	0–14
**Parent–child relationship ^f^**				
Total score (T3; 0–125)	14.23 (10.40)	0–102	13.43 (8.33)	0–43
Impaired bonding (T3; 0–60)	7.77 (5.83)	0–53	7.36 (4.93)	0–24
Rejection and anger (T3; 0–35)	3.87 (3.38)	0–32	3.49 (2.71)	0–14
Anxiety about care (T3; 0–20)	2.51 (2.02)	0–15	2.53 (1.67)	0–10
Risk of abuse (T3; 0–10)	0.08 (0.41)	0–5	0.04 (0.21)	0–1
**Child age in weeks** (T2)	8.38 (1.83)	4–16	8.81 (1.90)	5–16
**Child age in months** (T3)	13.73 (0.57)	12–16	13.86 (0.51)	13–16
**Child age in months** (T4)	23.91 (0.41)	23–25	24.00 (0.39)	23–25
**Child temperament** ^g^ (T2; 0–63)	28.79 (7.93)	10–54	28.77 (7.74)	11–54
**Child development ^h^**				
Total score (T4; 0–300)	260.96 (26.93)	0–300	256.66 (30.83)	0–300
Communication (T4; 0–60)	54.30 (9.34)	0–60	54.88 (8.46)	0–60
Gross motor (T4; 0–60)	54.39 (8.00)	0–60	53.96 (8.07)	0–60
Fine motor (T4; 0–60)	53.32 (6.62)	0–60	52.21 (7.35)	0–60
Problem-solving (T4; 0–60)	47.56 (9.64)	0–60	45.75 (10.50)	0–60
Personal-social (T4; 0–60)	51.33 (7.20)	0–60	49.87 (8.29)	0–60
	
	*n ^a^*	% ^b^	*n ^a^*	% ^b^
	
**Preterm birth ^i^ (T2)**				
Yes	25	3.7	14	3.2
No	649	96.3	428	96.8
**Parental country of birth** (T1)				
Germany	648	96.6	427	97.5
Other	23	3.4	11	2.5
**Parental academic degree (T1)**				
Completed university degree	414	61.5	257	59.4
No completed university degree	259	38.5	176	40.6
**Parental employment status ^j^ (T1)**				
Full-time employed	302	44.81	364	82.4
Part-time employed	127	18.84	38	8.6
Unemployed	10	1.5	3	0.7
Parental leave	92	13.6	1	0.2
**Parental relationship status (T1)**				
Committed relationship	668	99.6	437	99.8
No committed relationship	3	0.4	1	0.2
**Parental parity (T1)**				
Primiparous	538	80.4	347	81.3
Multiparous	131	19.6	80	18.7

T1, during pregnancy; T2, 8 weeks after anticipated birth date; T3, 14 months after the actual birth date; T4, 24 months after the actual birth date.

^a^n varies slightly due to missing data of some participants.

^b^Valid percent.

^c^Obsessive-compulsive subscale of the Symptom-Checklist Revised (SCL-90-R).

^d^EPDS, Edinburgh Postnatal Depression Scale.

^e^Anxiety subscale of the SCL-90-R.

^f^PBQ, Postpartum Bonding Questionnaire.

^g^ICQ, Fussy/difficult subscale of the Infants Characteristics Questionnaire.

^h^ASQ-3, 24-months version of the Ages and Stages Questionnaire-3.

^i^Preterm birth, born before the 37th week of gestation.

^j^Multiple answers possible.

### Dropout analyses

For both mothers and fathers, dropout analyses were performed to examine whether completers (T1–T4 completed) differed significantly from non-completers (T4 not completed) with respect to sociodemographic characteristics, predictor, mediator, and/or potential confounding variables (tables on request). Among fathers, completers were significantly more often first-time fathers compared to non-completers [81.26% vs. 69.86%; χ^2^(1) = 5.00, *p* = 0.025]. In terms of all other variables, there were no significant differences between completing and non-completing mothers and fathers.

### Regression analyses

**FIGURE 3:**
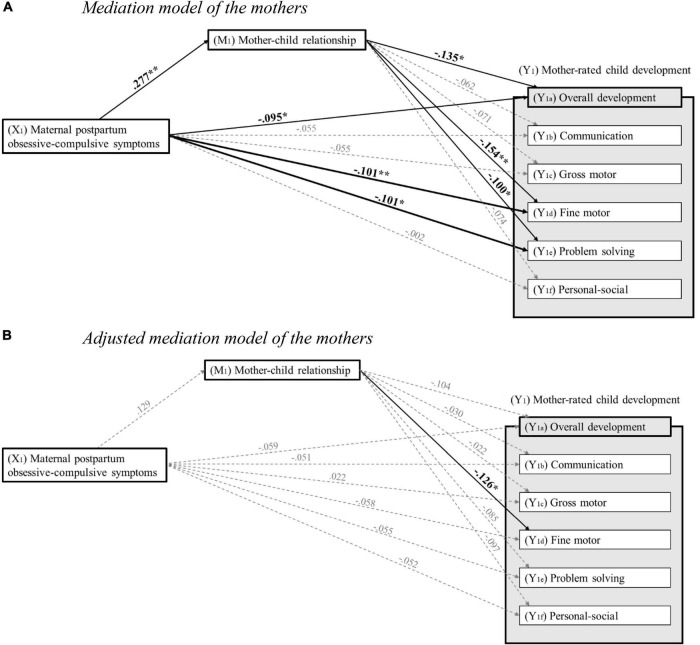
**(A)** Mediation model of the mothers. Standardized regression coefficients for the associations between maternal postpartum obsessive-compulsive symptoms 8 weeks after childbirth, mother–child relationship 14 months after childbirth, and child development 24 months after childbirth. *p < 0.05; **p < 0.01 (two-tailed testing). **(B)** Adjusted mediation model of the mothers. Standardized regression coefficients for the associations between maternal postpartum obsessive-compulsive symptoms 8 weeks after childbirth, mother–child relationship 14 months after childbirth, and child development 24 months after childbirth, adjusted for age, academic degree, postpartum depressive and anxiety symptoms of the mother, preterm birth and temperament of the child, as well as COVID-19 pandemic-driven adversities. *p < 0.05; **p < 0.01 (two-tailed testing).

**FIGURE 4:**
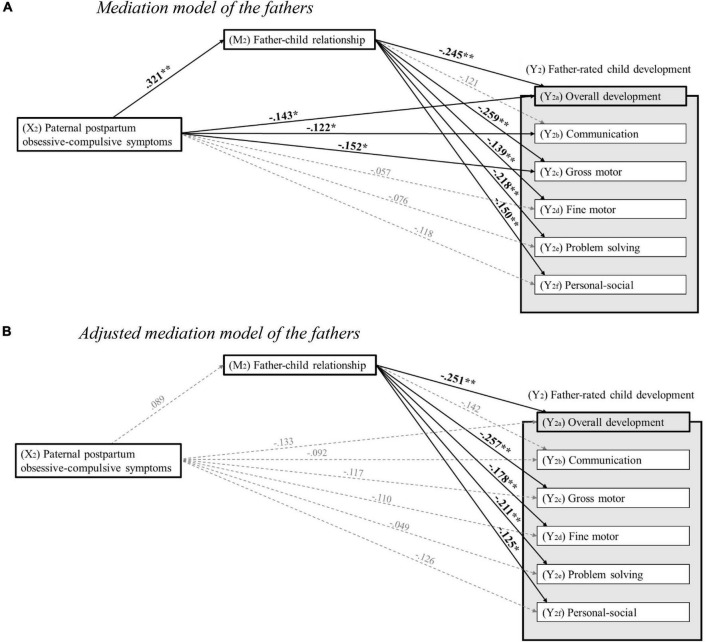
**(A)** Mediation model of the fathers. Standardized regression coefficients for the associations between paternal postpartum obsessive-compulsive symptoms 8 weeks after childbirth, father–child relationship 14 months after childbirth, and child development 24 months after childbirth. *p < 0.05; **p < 0.01 (two-tailed testing). **(B)** Adjusted mediation model of the fathers. Standardized regression coefficients for the associations between paternal postpartum obsessive-compulsive symptoms 8 weeks after childbirth, father–child relationship 14 months after childbirth, and child development 24 months after childbirth, adjusted for age, academic degree, postpartum depressive and anxiety symptoms of the father, preterm birth and temperament of the child, as well as COVID-19 pandemic-driven adversities. *p < 0.05; **p < 0.01 (two-tailed testing).

First, bivariate correlation analyses including all predictor, mediator, outcome, and potential confounding variables were conducted to explore the strength and direction of their respective associations (see Tables 2A,B). Subsequently, to investigate the prospective associations between parental postpartum obsessive-compulsive symptoms, parent–child relationship, and overall as well as domain-specific child development, linear regression analyses were conducted. For an overview illustrating the significant and non-significant associations, see Figures 3, 4. Tables showing the full results of the conducted analyses are provided in Supplementary material A.

**TABLE 2A:** Pearson’s correlation coefficients (*r*) including the predictor, mediator, outcome, and potential confounding variables for mothers.

	1.	2.	3.	4.	5.	6.	7.	8.	9.	10.	11.	12.	13.	14.	15.	
1.	Maternal postpartum obsessive-compulsive symptoms ^a^	–														
2.	Mother–child relationship ^b^	**0.277** **	–													
3.	Overall child development ^c^	**−0.095** *	**−0.135** **	–												
4.	Communication development ^c^	–0.055	–0.062	**0.644** **	–											
5.	Gross motor development ^c^	–0.055	–0.071	**0.622** **	**0.234** **	–										
6.	Fine motor development ^c^	**−0.101** **	**−0.154** **	**0.639** **	**0.228** **	**0.239** **	–									
7.	Problem-solving development ^c^	**−0.101** **	−0.100*	**0.702** **	**0.180** **	**0.304** **	**0.437** **	–								
8.	Personal-social development ^c^	0.002	–0.074	**0.687** **	**0.408** **	**0.290** **	**0.329** **	**0.309** **	–							
9.	Preterm birth ^d^	–0.026	0.012	–0.071	–0.048	–0.059	–0.072	–0.020	–0.044	–						
10.	Child temperament ^e^	0.095*	**0.267** **	–0.065	–0.019	–0.059	–0.075	–0.051	–0.016	0.013	–					
11.	Maternal age	0.047	–0.036	–0.030	0.003	–0.034	–0.043	–0.007	–0.025	0.082*	0.041	–				
12.	Maternal academic degree	–0.003	**0.132** **	0.037	0.023	–0.011	–0.008	0.065	0.036	–0.071	**0.157** **	**0.190** **	–			
13.	Maternal postpartum depressive symptoms ^f^	**0.524** **	**0.379** **	**−0.121** **	−0.088*	**−0.119** **	**−0.114** **	−0.092*	0.016	0.079*	**0.232** **	0.014	–0.033	–		
14.	Maternal postpartum anxiety symptoms ^g^	**0.593** **	**0.203** **	–0.073	–0.012	−0.090*	−0.095*	−0.100*	0.063	0.033	0.058	0.047	–0.047	**0.550** **	–	
15.	COVID-19 pandemic adversities	–	–	–0.021	–0.046	0.007	0.023	0.027	–0.065	–	–	–	–	–	–

Coefficients in bold are statistically significant.

^a^Obsessive-compulsive subscale of the Symptom-Checklist Revised (SCL-90-R).

^b^PBQ, Postpartum Bonding Questionnaire.

^c^ASQ-3, 24-months version of the Ages and StagesQuestionnaire-3.

^d^Born before the 37th week of gestation.

^e^ICQ, Fussy/difficult subscale of the Infants Characteristics Questionnaire.

^f^EPDS, Edinburgh Postnatal Depression Scale.

^g^Anxiety subscale of the SCL-90-R.

*p < 0.05; **p < 0.01 (two-tailed testing).

**TABLE 2B:** Pearson’s correlation coefficients (*r*) including the predictor, mediator, outcome, and potential confounding variables for fathers.

	1.	2.	3.	4.	5.	6.	7.	8.	9.	10.	11.	12.	13.	14.	15.	
1.	Paternal postpartum obsessive-compulsive symptoms ^a^	–														
2.	Father–child relationship ^b^	0.321**	–													
3.	Overall child development ^c^	**−0.143** **	**−0.245** **	–												
4.	Communication development ^c^	−0.122*	−0.121*	**0.679** **	–											
5.	Gross motor development ^c^	**−0.152** **	**−0.259** **	**0.672** **	**0.351** **	–										
6.	Fine motor development ^c^	–0.057	**−0.139** **	**0.701** **	**0.296** **	**0.316** **	–									
7.	Problem-solving development ^c^	–0.076	**−0.218** **	**0.785** **	**0.340** **	**0.382** **	**0.547** **	–								
8.	Personal-social development ^c^	−0.118*	**−0.150** **	**0.752** **	**0.458** **	**0.403** **	**0.408** **	**0.445** **	–							
9.	Preterm birth ^d^	–0.014	–0.004	0.013	0.018	–0.025	0.034	0.046	–0.011	–						
10.	Child temperament ^e^	**0.184** **	**0.388** **	**−0.134** **	–0.010	**−0.117** *	–0.047	**−0.145** **	**−0.153** **	–0.037	–					
11.	Paternal age	–0.026	–0.081	–0.059	0.034	–0.058	–0.021	–0.079	–0.065	0.001	–0.011	–				
12.	Paternal academic degree	–0.053	0.118	–0.091	0.065	–0.108	–0.081	–0.099	–0.107	–0.061	0.088	0.123	–			
13.	Paternal postpartum depressive symptoms ^f^	**0.612** **	**0.349** **	**−0.117** *	**−0.111** *	**−0.114** *	0.011	–0.079	**−0.124** *	0.054	**0.238** **	–0.009	–0.003	–		
14.	Paternal postpartum anxiety symptoms ^g^	**0.691** **	**0.307** **	–0.079	–0.072	−0.120*	–0.017	–0.039	–0.039	0.001	**0.128** **	0.061	–0.044	**0.541** *	–	
15.	COVID-19 pandemic adversities	–	–	–0.029	–0.071	–0.029	0.023	0.012	–0.045	–	–	–	–	–	–

Coefficients in bold are statistically significant.

^a^Obsessive-compulsive subscale of the Symptom-Checklist Revised (SCL-90-R).

^b^PBQ, Postpartum Bonding Questionnaire.

^c^ASQ-3, 24-months version of the Ages and Stages Questionnaire-3.

^d^Born before the 37th week of gestation.

^e^ICQ, Fussy/difficult subscale of the Infants Characteristics Questionnaire.

^f^EPDS, Edinburgh Postnatal Depression Scale.

^g^Anxiety subscale of the SCL-90-R.

*p < 0.05; **p < 0.01 (two-tailed testing).

#### Association between maternal postpartum obsessive-compulsive symptoms and child development

For mothers, results indicated that higher levels of maternal postpartum obsessive-compulsive symptoms at T2 significantly predicted poorer overall development (β = −0.095, *p* = 0.015), poorer fine motor development (β = −0.101, *p* = 0.006), and poorer problem-solving development (β = −0.101, *p* = 0.019) at T4. However, communication development (β = 0.150, *p* = 0.150), gross motor development (β = −0.055, *p* = 0.141), and personal-social development (β = 0.002, *p* = 0.957) at T4 were not significantly predicted by maternal postpartum obsessive-compulsive symptoms at T2. Furthermore, when age, academic degree, postpartum depressive and anxiety symptoms of the mother, preterm birth and temperament of the child, as well as COVID-19 pandemic-driven adversities were included as potential confounders in the regression models, neither overall child development (β = −0.059, *p* = 0.245), communication development (β = −0.051, *p* = 0.281), gross motor development (β = 0.022, *p* = 0.650), fine motor development (β = −0.058, *p* = 0.214), problem-solving development (β = −0.055, *p* = 0.300), nor personal-social development (β = −0.052, *p* = 0.369) at T4 were significantly predicted by maternal postpartum obsessive-compulsive symptoms at T2.

#### Association between paternal postpartum obsessive-compulsive symptoms and child development

For fathers, results showed that higher levels of paternal postpartum obsessive-compulsive symptoms at T2 significantly predicted poorer overall development (β = −0.143, *p* = 0.033), poorer communication development (β = −0.122, *p* = 0.035), and poorer gross motor development (β = −0.152, *p* = 0.018) at T4. However, fine motor development (β = −0.057, *p* = 0.209), problem-solving development (β = −0.076, *p* = 0.176), and personal-social development (β = −0.118, *p* = 0.076) at T4 were not significantly predicted by paternal postpartum obsessive-compulsive symptoms at T2. Furthermore, when age, academic degree, postpartum depressive and anxiety symptoms of the father, preterm birth and temperament of the child, as well as COVID-19 pandemic-driven adversities were included as potential confounders in the regression models, neither overall child development (β = −0.133, *p* = 0.106), communication development (β = −0.092, *p* = 0.218), gross motor development (β = −0.117, *p* = 0.179), fine motor development (β = −0.110, *p* = 0.152), problem-solving development (β = −0.049, *p* = 0.545), nor personal-social development (β = −0.126, *p* = 0.103) at T4 were significantly predicted by paternal postpartum obsessive-compulsive symptoms at T2.

#### Association between maternal postpartum obsessive-compulsive symptoms and mother–child relationship

For mothers, results revealed that higher levels of maternal postpartum obsessive-compulsive symptoms at T2 were significantly associated with poorer mother–child relationship at T3 (β = 0.277, *p* < 0.001). However, when age, academic degree, postpartum depressive and anxiety symptoms of the mother, preterm birth and temperament of the child, as well as COVID-19 pandemic-driven adversities were included as potential confounders in the regression model, the aforementioned association was no longer significant (β = 0.129, *p* = 0.054).

#### Association between paternal postpartum obsessive-compulsive symptoms and father–child relationship

For fathers, results indicated that higher levels of paternal postpartum obsessive-compulsive symptoms at T2 were significantly associated with poorer father–child relationship at T3 (β = 0.321, *p* < 0.001). However, when age, academic degree, postpartum depressive and anxiety symptoms of the father, preterm birth and temperament of the child, as well as COVID-19 pandemic-driven adversities were included as potential confounders in the regression model, the aforementioned association was no longer significant (β = 0.089, *p* = 0.230).

#### Association between mother–child relationship and child development

For mothers, results showed that poorer mother–child relationship at T3 significantly predicted poorer overall child development (β = −0.135, *p* = 0.019), poorer fine motor development (β = −0.154, *p* = 0.004), and poorer problem-solving development (β = −0.100, *p* = 0.036) at T4. However, communication development (β = −0.062, *p* = 0.178), gross motor development (β = −0.071, *p* = 0.097), and personal-social development (β = −0.074, *p* = 0.131) at T4 were not significantly predicted by mother–child relationship at T3. When age, academic degree, postpartum depressive and anxiety symptoms of the mother, preterm birth and temperament of the child, as well as COVID-19 pandemic-driven adversities were included as potential confounders in the regression models, poorer mother–child relationship at T3 was still a significant predictor of poorer fine motor development (β = −0.126, *p* = 0.029) at T4. However, after adjusting for the aforementioned potential confounders, neither overall child development (β = −0.104, *p* = 0.080), communication development (β = −0.030, *p* = 0.552), gross motor development (β = −0.022, *p* = 0.630), problem-solving development (β = −0.085, *p* = 0.119), nor personal-social development (β = −0.097, *p* = 0.073) at T4 were significantly predicted by mother–child relationship at T3.

#### Association between father–child relationship and child development

For fathers, results revealed that poorer father–child relationship at T3 significantly predicted poorer overall child development (β = −0.245, *p* < 0.001), poorer gross motor development (β = −0.259, *p* < 0.001), poorer fine motor development (β = −0.139, *p* = 0.009), poorer problem-solving development (β = −0.218, *p* < 0.001), and poorer personal-social development (β = −0.150, *p* = 0.010) at T4. However, communication development (β = −0.121, *p* = 0.075) at T4 was not significantly predicted by father–child relationship at T3. When age, academic degree, postpartum depressive and anxiety symptoms of the father, preterm birth and temperament of the child, as well as COVID-19 pandemic-driven adversities were included as potential confounders in the regression models, poorer father–child relationship at T3 was still a significant predictor of poorer overall child development (β = −0.251, *p* < 0.001), poorer gross motor development (β = −0.257, *p* < 0.001), poorer fine motor development (β = −0.178, *p* = 0.017), poorer problem-solving development (β = −0.211, *p* = 0.001), and poorer personal-social development (β = −0.125, *p* = 0.020) at T4. However, after adjusting for the aforementioned potential confounders, communication development (β = −0.142, *p* = 0.074) at T4 was not significantly predicted by father–child relationship at T3.

### Mediation analyses

For both mothers and fathers, mediation analyses were carried out in order to determine whether the associations between parental postpartum obsessive-compulsive symptoms and overall as well as domain-specific child development are mediated by the parent–child relationship (for tables, see Supplementary material B).

#### The mediating role of the mother–child relationship

For mothers, results revealed a significant indirect effect of maternal postpartum obsessive-compulsive symptoms on overall child development (ab = −0.206, 95% CI = −0.424; −0.026) and fine motor development (ab = −0.061, 95% CI = −0.112; −0.019) through the mother–child relationship. Since the direct effects of maternal postpartum obsessive-compulsive symptoms on overall child development (B = −0.441, *p* = 0.097) and fine motor development (B = −0.105, *p* = 0.108) were not significant in the mediation analyses, it can be assumed that the associations are fully mediated through the mother–child relationship. For communication development (ab = −0.025, 95% CI = −0.084; 0.028), gross motor development (ab = −0.030, 95% CI = −0.079; 0.012), problem-solving development (ab = −0.053, 95% CI = −0.116; 0.005), and personal-social development (ab = −0.038, 95% CI = −0.091; 0.007), no significant indirect effects were found, indicating no significant mediating role of the mother–child relationship. Furthermore, when including age, academic degree, postpartum depressive and anxiety symptoms of the mother, preterm birth and temperament of the child, as well as COVID-19 pandemic-driven adversities as potential confounders in the mediation models, the results indicated that the mother–child relationship does not act as a mediating factor between maternal postpartum obsessive-compulsive symptoms and overall child development (ab = −0.079, 95% CI = −0.195; 0.024), communication development (ab = −0.008, 95% CI = −0.034; 0.024), gross motor development (ab = −0.0006, 95% CI = −0.031; 0.019), fine motor development (ab = −0.024, 95% CI = −0.054; 0.002), problem-solving development (ab = −0.021, 95% CI = −0.025; 0.004), or personal-social development (ab = −0.021, 95% CI = −0.050; 0.005).

#### The mediating role of the father–child relationship

For fathers, results indicated a significant indirect effect of paternal postpartum obsessive-compulsive symptoms on overall child development (ab = −0.615, 95% CI = −1.041; −0.283), gross motor development (ab = −0.167, 95% CI = −0.281; −0.075), fine motor development (ab = −0.093, 95% CI = −0.187; −0.019), problem-solving development (ab = −0.203, 95% CI = −0.342; −0.091), and personal-social development (ab = −0.096, 95% CI = −0.199; −0.019) through the father–child relationship. Since the direct effects of paternal postpartum obsessive-compulsive symptoms on overall child development (B = −0.643, *p* = 0.136), gross motor development (B = −0.178, *p* = 0.113), fine motor development (B = −0.027, *p* = 0.801), problem-solving development (B = −0.031, *p* = 0.831), and personal-social development (B = −0.183, *p* = 0.120) were not significant in the mediation analyses, it can be assumed that the associations are fully mediated through the father–child relationship. For communication development (ab = −0.068, 95% CI = −0.187; 0.031), no significant indirect effect was found, indicating no significant mediating role of the father–child relationship. Furthermore, when including age, academic degree, postpartum depressive and anxiety symptoms of the father, preterm birth and temperament of the child, as well as COVID-19 pandemic-driven adversities as potential confounders in the mediation models, the results indicated that the father–child relationship does not act as a mediating factor between paternal postpartum obsessive-compulsive symptoms and overall child development (ab = −0.197, 95% CI = −0.579; 0.121), communication development (ab = −0.032, 95% CI = −0.124; 0.017), gross motor development (ab = −0.055; 95% CI = −0.157; 0.035), fine motor development (ab = −0.034, 95% CI = −0.107; 0.017), problem-solving development (ab = −0.059, 95% CI = −0.180; 0.033), or personal-social development (ab = −0.026, 95% CI = −0.090; 0.014).

## Discussion

The present prospective longitudinal study aimed to explore the associations between parental postpartum obsessive-compulsive symptoms, the parent–child relationship, and early child development among families from the general population in Germany. In this context, we focused on the overall development of the 2-year-old children as well as on their age-specific development in five different domains, namely communication, gross motor, fine motor, problem-solving, and personal-social development. Furthermore, this study set out to determine whether the parent–child relationship mediates the associations between parental postpartum obsessive-compulsive symptoms and the respective child developmental outcomes. In this way, we intended to advance the knowledge concerning the etiology of developmental delays in young children which in turn could contribute to the development and improvement of tailored prevention and treatment approaches to foster an optimal, age-appropriate development in children.

### Associations between parental postpartum obsessive-compulsive symptoms and child development

Our results revealed that, without accounting for potential confounding variables, higher levels of maternal postpartum obsessive-compulsive symptoms 8 weeks after delivery significantly predicted poorer overall child development, poorer fine motor development, and poorer problem-solving development in their 2-year-old toddlers. Furthermore, without controlling for potential confounding variables, paternal postpartum obsessive-compulsive symptoms 8 weeks after childbirth significantly predicted poorer overall child development, poorer communication development, and poorer gross motor development in their 2-year-old children. However, after adjusting for a number of potential confounding variables, all significant associations disappeared. Thus, contrary to our first hypothesis neither higher maternal postpartum obsessive-compulsive symptoms nor higher paternal postpartum obsessive-compulsive symptoms predicted any poorer child developmental outcomes. While most previous research has focused on the effects of postpartum depressive and anxiety symptoms (49, 50) and, to a lesser extent, on postpartum anxiety symptoms (49), our findings provide the first scientific contribution concerning the specific impact of postpartum obsessive-compulsive symptoms on several developmental domains as well as on the overall development in early childhood. However, while there is ample evidence suggesting that postpartum depressive symptoms negatively affect children’s cognitive (e.g., 43, 44), motor (e.g., 43, 45), language (e.g., 46, 47), and social-emotional (e.g., 48) development, our results do not suggest that parental postpartum obsessive-compulsive symptoms have a similarly harmful impact on the age-appropriate development in young children. Hereby, our findings regarding postpartum obsessive-compulsive symptoms are in line with previous studies that have also failed to identify a direct negative impact of postpartum anxiety symptoms on the cognitive (60–62), motor (57, 62), or language (57, 62) development in young children.

A possible explanation for these findings might be the overlapping clinical pictures and high comorbidity between postpartum depressive, anxiety, and obsessive-compulsive symptoms (100). Another possible explanation could be that only severe parental postpartum obsessive-compulsive symptoms affect offspring development, while our sample tended to experience mild postpartum obsessive-compulsive symptoms. Also, there is a possibility that not parental postpartum obsessive-compulsive symptoms *per se* are linked to poorer child development, but the frequently accompanying parental avoidance behavior, as already described in another study on postpartum anxiety symptoms and child development (60). However, parental avoidance behavior was not explicitly investigated in the current study and therefore its potential contribution to poorer child developmental outcomes needs further clarification.

### Associations between parental postpartum obsessive-compulsive symptoms and the parent–child relationship

Without considering potential confounding variables, we found that higher maternal postpartum obsessive-compulsive symptoms 8 weeks after delivery predicted poorer mother–child relationship 14 months after childbirth. In addition, without adjusting for potential confounding variables, higher levels of paternal postpartum obsessive-compulsive symptoms 8 weeks after childbirth similarly predicted poorer father–child relationship 14 months after childbirth. However, after controlling for a number of potential confounding variables, both of the aforementioned associations were no longer significant, which is contrary to our second hypothesis. With regard to the effects of postpartum mental health issues on the early parent–child relationship, most previous studies have focused on postpartum depressive symptoms, and, to a lesser extent, on postpartum anxiety symptoms. Hereby, our study adds valuable knowledge to this field of research by focusing on the largely understudied effects of parental postpartum obsessive-compulsive symptoms on the early parent–child relationship. However, while previous studies have repeatedly shown that both higher depressive symptom burden (e.g., 36–41) as well as higher anxiety symptom burden (36, 38, 40, 51–55) in the postpartum period are associated with a deterioration in the early parent–child relationship, our findings suggest that parental postpartum obsessive-compulsive symptoms do not have a comparable adverse effect on parental bonding quality. These findings are somewhat surprising given the fact that obsessions and compulsions in the postpartum period often revolve around the health and well-being of the newborn child (68, 70, 75–77) and that other studies have demonstrated that mothers experiencing postpartum obsessive-compulsive symptoms are less sensitive in their interaction behavior with their infants compared to healthy mothers (65, 101). Indeed, a recent population-based study among Israeli women indicates that maternal postpartum obsessive-compulsive symptoms are associated with poorer mother–child relationship 4 months after delivery (102). One possible explanation for this unexpected result could be the fact that Ratzoni et al. focused on a specific subtype/manifestation of postpartum obsessive-compulsive disorder, namely parent–child relationship obsessive-compulsive disorder. Furthermore, the high comorbidity between postpartum depressive, anxiety, and obsessive-compulsive symptoms as well as the rather low symptom burden in the postpartum period might also explain our findings. However, the extent to which distinct effects of parental postpartum obsessive-compulsive symptoms become apparent only at a higher severity level requires further investigation. Last but not least, it again remains unclear to what extent parental postpartum obsessive-compulsive symptoms actually have a direct effect on parent–child relationship, or whether a poorer parent–child relationship could be rather explained by parental avoidance behavior that is often linked to postpartum obsessive-compulsive symptoms (65–68).

### Associations between parent–child relationship and child development

Another major finding to emerge from the present study is that, without accounting for potential confounding variables, poorer mother–child relationship 14 months postpartum significantly predicted poorer overall development, poorer fine motor development, and poorer problem-solving development in their 2-year-old children. In addition, poorer father–child relationship 14 months after childbirth was a significant predictor of poorer overall child development, poorer gross motor development, poorer fine motor development, poorer problem-solving development, and poorer personal-social development in their 2-year-old toddlers. After adjusting for a range of potential confounders, poorer mother–child relationship 14 months postpartum still significantly predicted poorer fine motor development in their 2-year-old toddlers. Furthermore, after controlling for a number of potential confounders, poorer father–child relationship 14 months postpartum still significantly predicted poorer overall development, poorer gross motor development, poorer fine motor development, poorer problem-solving development, and poorer personal-social development in their 2-year-old children. Accordingly, we were able to partially confirm our third hypothesis. With the present study, we provide an important scientific contribution to the still understudied associations between parent–child relationship, especially in the postpartum period, and early child developmental outcomes. According to a meta-analysis by Le Bas et al. (103), although several studies have examined the interplay between prepartum mother-fetus bonding and early infant temperament, only few studies to date have shed light on the associations between postpartum parent–child relationship and other important domains of child development. To date, there are only two studies in this area that have examined the association between postpartum mother–child relationship and the child’s social-emotional development (39, 104). A large-scale prospective study from Australia has shown that poorer mother–child-relationship at 8 weeks after childbirth is associated with poorer social-emotional development in their 12-month-old infants (104). Moreover, another study revealed that a mother’s felt bond toward her baby acts as a mediating factor linking maternal postpartum depressive symptoms 2 months after childbirth to the social-emotional development in her 6-month-old infant (39). Other domains, such as motor, language, or cognitive child development have been largely neglected so far. In addition, the few existing studies merely focused on the mother–child relationship and generally overlooked the potential impact of the father–child relationship on early child development. Furthermore, there is a dearth of large-scale prospective longitudinal studies addressing this crucial topic (103), making it difficult to compare our findings with previous studies. Thus, given the paucity of evidence, further research is needed to replicate our findings, especially longitudinal studies investigating the impact of both mother–child relationship and father–child relationship on different domains of child development. In this context, it seems to be particularly relevant that future studies also account for gender differences in both parents and their offspring in order to investigate potential gender differences in greater detail. Based on previous studies, it is evident that parents tend to behave differently toward their daughters and sons (105), which can be attributed to both neurobiological (106) and cultural factors (107). As a consequence, it seems to be of high importance to take both the parents’ and their offspring’s gender into account, as these factors might have reciprocal effects on parent–child relationships (108), which in turn might have an impact on different domains of child development.

### The mediating role of the parent–child relationship

Finally, the results of the present study revealed that, without accounting for potential confounding variables, the mother–child relationship 14 months postpartum fully mediates the associations between maternal postpartum obsessive-compulsive symptoms 8 weeks after childbirth and overall child development as well as fine motor development 24 months after childbirth. Moreover, without controlling for potential confounding variables, the father–child relationship 14 months postpartum fully mediates the associations between paternal postpartum obsessive-compulsive symptoms 8 weeks after childbirth and overall child development, gross motor development, fine motor development, problem-solving development, and personal-social development 24 months after childbirth. However, after controlling for a range of potential confounders neither the mother–child relationship nor the father–child relationship was a significant mediating factor between parental postpartum obsessive-compulsive symptoms and any child developmental outcomes, which is contrary to our fourth hypothesis. While Mason et al. (39) found that the mother–child relationship mediates the association between maternal postpartum depressive symptoms and early social-emotional child development, our results suggest that such a mediating role between parental postpartum obsessive-compulsive symptoms and early child development does not exist when other potential confounding factors are considered. However, again, it remains unclear whether these findings are due to the high comorbidity between postpartum depressive, anxiety, and obsessive-compulsive symptoms as well as the low symptom burden in our present sample. Hence, it is possible that distinct mediating effects only become apparent at a certain level of severity.

### Strengths and limitations

The current study has several major strengths. First, while most studies to date focused on the effects of postpartum depressive and anxiety symptoms, this is the first study to examine the prospective longitudinal associations between parental postpartum obsessive-compulsive symptoms, parent–child relationship, and child development. Second, our results are based on the analysis of a large population-based sample. Third, controlling for important parent- and child-related potential confounding variables as well as COVID-19 pandemic-driven adversities provided the opportunity to determine the unique prospective longitudinal associations between parental postpartum obsessive-compulsive symptoms, the parent–child relationship, and child development. Fourth, by including both mothers and fathers, it was possible to examine differential influences of both parents on the respective parent–child relationship and child developmental outcomes. Fifth, through the investigation of five major domains of child development as well as the overall child development, we were able to gain a comprehensive overview of the effects of parental postpartum obsessive-compulsive symptoms and early parent–child relationship on different domains of child development. Sixth, all factors under investigation were prospectively collected, which reduces a potential recall bias. Finally, our long study period of 2 years covers an important period of early child development.

Despite these strengths, however, this study is also subject to limitations. First, it should be mentioned that our sample consisted of predominately well-educated German mothers and fathers who were living in a permanent partnership and expecting their first child. For this reason, there is a risk of self-selection bias. Given that a high proportion of our sample thus should be classified as socio-economically well-situated first-time parents, one should be careful to generalize these results to other populations, such as lower-educated parents, parents with more children, single parents without social support, or parents with a migration background and/or different cultural background. Although these groups of people are represented to a small percentage in the present study, it might be worthwhile to specifically investigate these populations in future studies in order to draw differential conclusions. Second, even though we used widely established instruments, our results are based on parental self-report questionnaires, which bears the risk of a bias due to social desirability. In addition, parental self-report questionnaires are highly subjective. Therefore, in order to gain a more objective rating of the parent–child relationship as well as the child development, future studies should also use independent observers and standardized assessment methods (e.g., the Bayley Scales of Infant and Toddler Development (109) to assess the child development as well as techniques of direct observation of the interactions in the significant contexts of feeding and play to assess the parent–child relationship). Third, in this context it should also be mentioned that while the reliability of the total score of the ASQ-3 was good to very good in the present study, the reliabilities of the domain-specific scores of child development were rather moderate to poor. Thus, the obtained results here should also be interpreted rather tentatively and require replication in future studies. Fourth, it should be kept in mind that the obsessive-compulsive subscale of the SCL-90-R was not specifically designed to screen for postpartum psychopathological symptoms and does not include questions about infant-related intrusive thoughts or parental avoidance behavior, which are quite common in the postpartum period. Consequently, there is a risk that affected parents may not have recognized their obsessions in the rather unspecific questions. Fifth, it is important to note that both the mothers and fathers in our sample were experiencing rather low symptom burden in the early postpartum period and reported, on average, a good parent–child relationship. For this reason, our results are likely rather less transferable to clinical populations with full-blown obsessive-compulsive disorder or parents with marked disturbances in the parent–child relationship.

### Implications for clinical practice and future research

Several implications for clinical practice and future research emerge from the present findings. Given that our results have demonstrated that not only a poorer mother–child relationship but also a poorer father–child relationship may have a direct negative impact on specific domains of child development, it seems urgently indicated to include both parents in future research as well as in clinical practice, e.g., by developing family-based interventions to strengthen the parent–child relationship. In addition, the extent to which mothers and fathers actually have differential influences on different domains of child development should be investigated in future epidemiological and clinical studies. Furthermore, it might be interesting to replicate these findings in parents with more pronounced difficulties in the parent–child relationship and to investigate the potential impact of significant others, such as grandparents or siblings. Furthermore, although our results do not suggest that higher levels of postpartum obsessive-compulsive symptoms have a negative impact on the parent–child relationship or early child development over and above other psychopathology, the present study may stimulate future research in the previously understudied field of parental postpartum obsessive-compulsive symptoms. In this context, it seems of particular relevance to investigate the potential adverse effects of full-blown obsessive-compulsive disorder in the postpartum period on the parent–child relationship and child development, ideally by using instruments designed for the postpartum period screening for specific infant-related obsessions and compulsions (e.g., the Parental Thoughts and Behaviors Checklist; 110). Keeping in mind that postpartum depressive, anxiety, and obsessive-compulsive symptoms tend to be highly comorbid, it might also be interesting to further investigate the potential adverse impact of transdiagnostic risk factors, such as parental avoidance behavior. Last but not least, future studies should replicate our obtained results in more diverse populations, such as parents with more children, less-educated parents, single parents, LGBTQ+ parents, as well as parents with different cultural background, ideally by using a multi-method approach and accounting for dyadic effects [e.g., by using the Actor-Partner Interdependence Model (111, 112)]. Although these populations were represented to a small proportion in our study, by including a larger number of people with different characteristics and personal circumstances of life, it would be possible to compare different groups of people in order to draw more specific conclusions with regard to the research questions under investigation.

## Conclusion

Previous research has shown that both postpartum depressive and anxiety symptoms may have a negative impact on the parent–child relationship as well as early child development. The present study advances the scientific knowledge in the field of peripartum research by shedding light on the yet understudied longitudinal associations between postpartum obsessive-compulsive symptoms, the parent–child relationship, and early child developmental outcomes. In this study, neither maternal nor paternal postpartum obsessive-compulsive symptoms 8 weeks after childbirth were found to have an adverse effect on the respective parent–child relationship 14 months postpartum when adjusting for potential confounders. Likewise, neither maternal nor paternal postpartum obsessive-compulsive symptoms 8 weeks after childbirth had a negative influence on the child development of their 2-year-old toddlers when adjusting for potential confounders. A previously hypothesized mediating role of the parent–child relationship between parental postpartum obsessive-compulsive symptoms and child development could not be confirmed. However, given that our sample reported a rather low level of obsessive-compulsive symptom burden in the postpartum period, future studies should examine whether the presumed associations only become apparent in full-blown postpartum obsessive-compulsive disorder. Furthermore, this study revealed that a poorer mother–child relationship 14 months after delivery was related to poorer fine motor development in their 2-year-old children. Also, a poorer father–child relationship 14 months after childbirth predicted both a poorer overall development as well as poorer gross motor, fine motor, problem-solving, and personal-social development in their 2-year-old children. As a result, it seems to be of urgent relevance to address both parents in clinical practice and research and not to focus solely on the mother–child dyad. In addition, the extent to which mothers and fathers actually have differential influences on different developmental domains should be investigated in future studies.

## Data availability statement

The datasets presented in this article are not readily available because of legal and ethical constraints. Public sharing of participant data was not included in the informed consent of the study. Requests to access the datasets should be directed to SG-N, susan.garthus-niegel@uniklinikum-dresden.de.

## Ethics statement

This study involving human participants was reviewed and approved by the Ethics Committee of the Medical Faculty of the Technische Universität Dresden. All participants provided their written informed consent to participate in this study.

## Author contributions

SB and SG-N developed the research questions. SB performed the statistical analyses and wrote the manuscript under the supervision of SG-N. JTM, VW, and MK supported the conduction of the study and prepared the data for the statistical analyses. EA and JM contributed with their expertise in this research field. SG-N acquired the funding, was responsible for the conception and design of the basic DREAM study with its sub-studies as well as the coordination and supervision of the data collection and the ongoing cohort study. All authors contributed to manuscript revision, read, and approved the submitted version.
