# Supplementary material for: The impact of postpartum obsessive-compulsive symptoms on child development and the mediating role of the parent–child relationship: A prospective longitudinal study
Source: Front Psychiatry. 2022 Aug 16;13:886347. doi: 10.3389/fpsyt.2022.886347 (PMC9532008; doi:10.3389/fpsyt.2022.886347)
Supplement: Supplementary file 1 [file Table_1.DOCX]

**Supplementary Material A: Full results of the regression analyses**

**Supplementary Table A.1a**

*Predictive value of maternal PPOCS^*^ on overall child development (model 1), and adjusting for seven potential confounding variables (model 2)*

|  | | Overall child development | | | | |
| --- | --- | --- | --- | --- | --- | --- |
| Model | Predictors | *B* | *SE* | ß | *p* | BCa 95% CI |
| 1 | Maternal PPOCS | -0.622 | 0.251 | -.095 | .015 | [-1.132; 0.141] |
| 2 | Maternal PPOCS | -0.386 | 0.335 | -.059 | .245 | [-1.068; 0.251] |
|  | Maternal age | -0.204 | 0.303 | -.029 | .488 | [-0.772; 0.385] |
|  | Maternal education | 2.265 | 2.214 | .041 | .312 | [-1.913; 6.456] |
|  | Maternal PPDS*^**^* | -0.664 | 0.383 | -.092 | .086 | [-1.436; 0.064] |
|  | Maternal PPAS*^***^* | 0.191 | 0.499 | .019 | .696 | [-0.737; 1.213] |
|  | Preterm birth | -10.270 | 8.350 | -.072 | .213 | [-28.538; 5.517] |
|  | Child temperament | -0.153 | 0.133 | -.045 | .252 | [-0.416; 0.106] |
|  | COVID-19 pandemic | -0.686 | 2.369 | -.011 | .774 | [-5.325; 4.043] |

*Note.* Simple and multiple linear regression analyses based on 5,000 bootstrapping iterations. In Model 2 forced entry was used.

Model 1: adjusted R^2^ = .008, *F*(1,652) = 5.936, *p* = .015; Model 2: adjusted R^2^ = .015, *F*(8,631) = 2.238, *p* = .023

B = unstandardized beta coefficient. SE = standard error for unstandardized beta; ß = standardized beta coefficient;
*p* = bootstrapped p-value; BCa 95% CI = Bias corrected and accelerated 95% confidence interval with α = .05

*^*^*postpartum obsessive-compulsive symptoms

*^**^*postpartum depression symptoms

*^***^*postpartum anxiety symptoms

**Supplementary Table A.1b**

*Predictive value of maternal PPOCS^*^ on communication development (model 1), and adjusting for seven potential confounding variables (model 2)*

|  | | Communication development | | | | |
| --- | --- | --- | --- | --- | --- | --- |
| Model | Predictors | *B* | *SE* | ß | *p* | BCa 95% CI |
| 1 | Maternal PPOCS | -0.124 | 0.086 | -.055 | .150 | [-0.302; 0.046] |
| 2 | Maternal PPOCS | -0.117 | 0.108 | -.051 | .281 | [-0.331; 0.094] |
|  | Maternal age | 0.006 | 0.107 | .003 | .959 | [-0.209; 0.223] |
|  | Maternal education | 0.420 | 0.784 | .022 | .598 | [-1.028; 1.990] |
|  | Maternal PPDS*^**^* | -0.245 | 0.152 | -.098 | .108 | [-0.558; 0.065] |
|  | Maternal PPAS*^***^* | 0.278 | 0.178 | .079 | .112 | [-0.084; 0.645] |
|  | Preterm birth | -2.068 | 2.712 | -.042 | .443 | [-8.483; 2.995] |
|  | Child temperament | -0.002 | 0.045 | -.001 | .972 | [-0.091; 0.090] |
|  | COVID-19 pandemic | -0.845 | 0.828 | -.040 | .314 | [-2.376; 0.703] |

*Note.* Simple and multiple linear regression analyses based on 5,000 bootstrapping iterations. In Model 2 forced entry was used.

Model 1: adjusted R^2^ = .001 , *F*(1,654) = 1.955, *p* = .162; Model 2: adjusted R^2^ = .003, *F*(8,633) = 1.205, *p* = .293

B = unstandardized beta coefficient. SE = standard error for unstandardized beta; ß = standardized beta coefficient;
*p* = bootstrapped p-value; BCa 95% CI = Bias corrected and accelerated 95% confidence interval with α = .05

*^*^*postpartum obsessive-compulsive symptoms

*^**^*postpartum depression symptoms

*^***^*postpartum anxiety symptoms

**Supplementary Table A.1c**

*Predictive value of maternal PPOCS^*^ on gross motor development (model 1), and adjusting for seven potential confounding variables (model 2)*

|  | | Gross motor development | | | | |
| --- | --- | --- | --- | --- | --- | --- |
| Model | Predictors | *B* | *SE* | ß | *p* | BCa 95% CI |
| 1 | Maternal PPOCS | -0.106 | 0.072 | -.055 | .141 | [-0.251; 0.038] |
| 2 | Maternal PPOCS | 0.043 | 0.096 | .022 | .650 | [-0.147; 0.234] |
|  | Maternal age | -0.052 | 0.083 | -.025 | .528 | [-0.219; 0.123] |
|  | Maternal education | -0.271 | 0.659 | -.016 | .689 | [-1.548; 1.088] |
|  | Maternal PPDS*^**^* | -0.222 | 0.122 | -.103 | .068 | [-0.490; 0.024] |
|  | Maternal PPAS*^***^* | -0.137 | 0.164 | -.045 | .400 | [-0.464; 0.174] |
|  | Preterm birth | -2.530 | 2.816 | -.060 | .369 | [-9.011; 2.775] |
|  | Child temperament | -0.024 | 0.042 | -.024 | .567 | [-0.103; 0.055] |
|  | COVID-19 pandemic | 0.076 | 0.713 | .004 | .916 | [-1.280; 1.439] |

*Note.* Simple and multiple linear regression analyses based on 5,000 bootstrapping iterations. In Model 2 forced entry was used.

Model 1: adjusted R^2^ = .001, *F*(1,654) = 1.953, *p* = .163; Model 2: adjusted R^2^ = .010, *F*(8,633) = 1.791, *p* = .076

B = unstandardized beta coefficient. SE = standard error for unstandardized beta; ß = standardized beta coefficient;
*p* = bootstrapped p-value; BCa 95% CI = Bias corrected and accelerated 95% confidence interval with α = .05

*^*^*postpartum obsessive-compulsive symptoms

*^**^*postpartum depression symptoms

*^***^*postpartum anxiety symptoms

**Supplementary Table A.1d**

*Predictive value of maternal PPOCS^*^ on fine motor development (model 1), and adjusting for seven potential confounding variables (model 2)*

|  | | Fine motor development | | | | |
| --- | --- | --- | --- | --- | --- | --- |
| Model | Predictors | *B* | *SE* | ß | *p* | BCa 95% CI |
| 1 | Maternal PPOCS | -0.162 | 0.060 | -.101 | .006 | [-0.277; -0.048] |
| 2 | Maternal PPOCS | -0.093 | 0.075 | -.058 | .214 | [-0.243; 0.056] |
|  | Maternal age | -0.058 | 0.077 | -.034 | .448 | [-0.203; 0.095] |
|  | Maternal education | -0.040 | 0.543 | -.003 | .941 | [-1.087; 1.033] |
|  | Maternal PPDS*^**^* | -0.143 | 0.092 | -.081 | .122 | [-0.332; 0.036] |
|  | Maternal PPAS*^***^* | -0.045 | 0.129 | -.018 | .730 | [-0.296; 0.213] |
|  | Preterm birth | -2.767 | 2.021 | -.079 | .165 | [-7.127; 1.275] |
|  | Child temperament | -0.038 | 0.034 | -.046 | .267 | [-0.108; 0.031] |
|  | COVID-19 pandemic | 0.382 | 0.594 | .025 | .518 | [-0.770; 1.559] |

*Note.* Simple and multiple linear regression analyses based on 5,000 bootstrapping iterations. In Model 2 forced entry was used.

Model 1: adjusted R^2^ = .009, *F*(1,654) = 6.675, *p* = .010; Model 2: adjusted R^2^ = .019, *F*(8,633) = 2.559, *p* = .009

B = unstandardized beta coefficient. SE = standard error for unstandardized beta; ß = standardized beta coefficient;
*p* = bootstrapped p-value; BCa 95% CI = Bias corrected and accelerated 95% confidence interval with α = .05

*^*^*postpartum obsessive-compulsive symptoms

*^**^*postpartum depression symptoms

*^***^*postpartum anxiety symptoms

**Supplementary Table A.1e**

*Predictive value of maternal PPOCS^*^ on problem-solving development (model 1), and adjusting for seven potential confounding variables (model 2)*

|  | | Problem-solving development | | | | |
| --- | --- | --- | --- | --- | --- | --- |
| Model | Predictors | *B* | *SE* | ß | *p* | BCa 95% CI |
| 1 | Maternal PPOCS | -0.236 | 0.101 | -.101 | .019 | [-0.434; -0.036] |
| 2 | Maternal PPOCS | -0.127 | 0.123 | -.055 | .300 | [-0.369; 0.107] |
|  | Maternal age | -0.034 | 0.101 | -.014 | .735 | [-0.226; 0.169] |
|  | Maternal education | 1.376 | 0.817 | .070 | .094 | [-0.282; 2.995] |
|  | Maternal PPDS*^**^* | -0.071 | 0.124 | -.028 | .561 | [-0.323; 0.182] |
|  | Maternal PPAS*^***^* | -0.186 | 0.186 | -.052 | .311 | [-0.558; 0.197] |
|  | Preterm birth | -1.392 | 2.012 | -.028 | .483 | [-5.239; 2.373] |
|  | Child temperament | -0.061 | 0.048 | -.050 | .200 | [-0.155; 0.028] |
|  | COVID-19 pandemic | 0.783 | 0.891 | .036 | .381 | [-0.991; 2.510] |

*Note.* Simple and multiple linear regression analyses based on 5,000 bootstrapping iterations. In Model 2 forced entry was used.

Model 1: adjusted R^2^ = .009, *F*(1,652) = 6.754, *p* = .010; Model 2: adjusted R^2^ = .011, *F*(8,631) = 1.903, *p* = .057

B = unstandardized beta coefficient. SE = standard error for unstandardized beta; ß = standardized beta coefficient;
*p* = bootstrapped p-value; BCa 95% CI = Bias corrected and accelerated 95% confidence interval with α = .05

*^*^*postpartum obsessive-compulsive symptoms

*^**^*postpartum depression symptoms

*^***^*postpartum anxiety symptoms

**Supplementary Table A.1f**

*Predictive value of maternal PPOCS^*^ on personal-social development (model 1), and adjusting for seven potential confounding variables (model 2)*

|  | | Personal-social development | | | | |
| --- | --- | --- | --- | --- | --- | --- |
| Model | Predictors | *B* | *SE* | ß | *p* | BCa 95% CI |
| 1 | Maternal PPOCS | 0.004 | 0.067 | .002 | .957 | [-0.131; 0.138] |
| 2 | Maternal PPOCS | -0.092 | 0.103 | -.052 | .369 | [-0.297; 0.103] |
|  | Maternal age | -0.063 | 0.078 | -.034 | .414 | [-0.212; 0.094] |
|  | Maternal education | 0.793 | 0.614 | .054 | .198 | [-0.389; 1.988] |
|  | Maternal PPDS*^**^* | 0.014 | 0.101 | .007 | .884 | [-0.190; 0.214] |
|  | Maternal PPAS*^***^* | 0.279 | 0.154 | .103 | .062 | [-0.024; 0.587] |
|  | Preterm birth | -1.526 | 1.918 | -.040 | .409 | [-5.451; 1.974] |
|  | Child temperament | -0.028 | 0.036 | -.030 | .436 | [-0.098; 0.041] |
|  | COVID-19 pandemic | -1.066 | 0.661 | -.065 | .108 | [-2.321; 0.207] |

*Note.* Simple and multiple linear regression analyses based on 5,000 bootstrapping iterations. In Model 2 forced entry was used.

Model 1: adjusted R^2^ = -.002, *F*(1,654) = .003, *p* = .959; Model 2: adjusted R^2^ = .004, *F*(8,633) = 1.289, *p* = .246

B = unstandardized beta coefficient. SE = standard error for unstandardized beta; ß = standardized beta coefficient;
*p* = bootstrapped p-value; BCa 95% CI = Bias corrected and accelerated 95% confidence interval with α = .05

*^*^*postpartum obsessive-compulsive symptoms

*^**^*postpartum depression symptoms

*^***^*postpartum anxiety symptoms

**Supplementary Table A.2**

*Predictive value of maternal PPOCS^*^ on mother-child relationship (model 1), and adjusting for six potential confounding variables (model 2)*

|  | | Mother-child relationship | | | | |
| --- | --- | --- | --- | --- | --- | --- |
| Model | Predictors | *B* | *SE* | ß | *p* | BCa 95% CI |
| 1 | Maternal PPOCS | 0.704 | 0.129 | .277 | <.001 | [0.491; 0.962] |
| 2 | Maternal PPOCS | 0.329 | 0.172 | .129 | .054 | [0.026; 0.690] |
|  | Maternal age | -0.196 | 0.096 | -.072 | .045 | [-0.386; -0.008] |
|  | Maternal education | 2.649 | 0.799 | .123 | .001 | [1.117; 4.224] |
|  | Maternal PPDS*^**^* | 0.853 | 0.158 | .304 | <.001 | [0.551; 1.157] |
|  | Maternal PPAS*^***^* | -0.165 | 0.209 | -.042 | .423 | [-0.610; 0.244] |
|  | Preterm birth | 0.779 | 2.549 | .014 | .757 | [-3.573; 5.650] |
|  | Child temperament | 0.231 | 0.048 | .175 | <.001 | [0.138; 0.320] |

*Note.* Simple and multiple linear regression analyses based on 5,000 bootstrapping iterations. In Model 2 forced entry was used.

Model 1: adjusted R^2^ = .076, *F*(1,659) = 54.902, *p* < .001; Model 2: adjusted R^2^ = .202, *F*(7,640) = 24.330, *p* < .001

B = unstandardized beta coefficient. SE = standard error for unstandardized beta; ß = standardized beta coefficient;
*p* = bootstrapped p-value; BCa 95% CI = Bias corrected and accelerated 95% confidence interval with α = .05

*^*^*postpartum obsessive-compulsive symptoms

*^**^*postpartum depression symptoms

*^***^*postpartum anxiety symptoms

**Supplementary Table A.3a**

*Predictive value of mother-child relationship on overall development (model 1), and adjusting for seven potential confounding variables (model 2)*

|  | | Overall child development | | | | |
| --- | --- | --- | --- | --- | --- | --- |
| Model | Predictors | *B* | *SE* | ß | *p* | BCa 95% CI |
| 1 | Mother-child relationship | -0.349 | 0.146 | -.135 | .019 | [-0.659; -0.097] |
| 2 | Mother-child relationship | -0.267 | 0.155 | -.104 | .080 | [-0.603; 0.001] |
|  | Maternal age | -0.270 | 0.307 | -.039 | .376 | [-0.856; 0.380] |
|  | Maternal education | 2.772 | 2.136 | .050 | .199 | [-1.230; 6.851] |
|  | Maternal PPDS*^*^* | -0.517 | 0.374 | -.071 | .170 | [-1.304; 0.229] |
|  | Maternal PPAS*^**^* | -0.125 | 0.458 | -.012 | .783 | [-1.003; 0.823] |
|  | Preterm birth | -9.792 | 8.150 | -.069 | .228 | [-27.542; 5.562] |
|  | Child temperament | -0.085 | 0.138 | -.025 | .527 | [-0.355; 0.200] |
|  | COVID-19 pandemic | -0.465 | 2.355 | -.008 | .840 | [-5.305; 4.392] |

*Note.* Simple and multiple linear regression analyses based on 5,000 bootstrapping iterations. In Model 2 forced entry was used.

Model 1: adjusted R^2^ = .017, *F*(1,654) = 12.153, *p* = .001; Model 2: adjusted R^2^ = .022, *F*(8,626) = 2.799, *p* = .005

B = unstandardized beta coefficient. SE = standard error for unstandardized beta; ß = standardized beta coefficient;
*p* = bootstrapped p-value; BCa 95% CI = Bias corrected and accelerated 95% confidence interval with α = .05

*^*^*postpartum depression symptoms

*^**^*postpartum anxiety symptoms

**Supplementary Table A.3b**

*Predictive value of mother-child relationship on communication development (model 1), and adjusting for seven potential confounding variables (model 2)*

|  | | Communication development | | | | |
| --- | --- | --- | --- | --- | --- | --- |
| Model | Predictors | *B* | *SE* | ß | *p* | BCa 95% CI |
| 1 | Mother-child relationship | -0.055 | 0.041 | -.062 | .178 | [-0.139; 0.019] |
| 2 | Mother-child relationship | -0.026 | 0.044 | -.030 | .552 | [-0.117; 0.055] |
|  | Maternal age | 0.003 | 0.108 | .001 | .976 | [-0.212; 0.238] |
|  | Maternal education | 0.387 | 0.784 | .020 | .621 | [-1.128; 2.015] |
|  | Maternal PPDS*^*^* | -0.259 | 0.156 | -.104 | .097 | [-0.600; 0.076] |
|  | Maternal PPAS*^**^* | 0.175 | 0.182 | .050 | .327 | [-0.164; 0.503] |
|  | Preterm birth | -1.998 | 2.698 | -.041 | .465 | [-8.095; 3.103] |
|  | Child temperament | 0.004 | 0.046 | .003 | .934 | [-0.091; 0.094] |
|  | COVID-19 pandemic | -0.684 | 0.843 | -.032 | .412 | [-2.263; 1.003] |

*Note.* Simple and multiple linear regression analyses based on 5,000 bootstrapping iterations. In Model 2 forced entry was used.

Model 1: adjusted R^2^ = .002, *F*(1,657) = 2.498, *p* = .114; Model 2: adjusted R^2^ = .002, *F*(8,628) = 1.125, *p* = .344

B = unstandardized beta coefficient. SE = standard error for unstandardized beta; ß = standardized beta coefficient;
*p* = bootstrapped p-value; BCa 95% CI = Bias corrected and accelerated 95% confidence interval with α = .05

*^*^*postpartum depression symptoms

*^**^*postpartum anxiety symptoms

**Supplementary Table A.3c**

*Predictive value of mother-child relationship on gross motor development (model 1), and adjusting for seven potential confounding variables (model 2)*

|  | | Gross motor development | | | | |
| --- | --- | --- | --- | --- | --- | --- |
| Model | Predictors | *B* | *SE* | ß | *p* | BCa 95% CI |
| 1 | Mother-child relationship | -0.055 | 0.033 | -.071 | .097 | [-0.125; 0.004] |
| 2 | Mother-child relationship | -0.017 | 0.036 | -.022 | .630 | [-0.091; 0.045] |
|  | Maternal age | -0.059 | 0.082 | -.028 | .478 | [-0.219; 0.098] |
|  | Maternal education | -0.218 | 0.661 | -.013 | .744 | [-1.447; 1.072] |
|  | Maternal PPDS*^*^* | -0.188 | 0.124 | -.087 | .130 | [-0.473; 0.076] |
|  | Maternal PPAS*^**^* | -0.116 | 0.167 | -.038 | .485 | [-0.435; 0.211] |
|  | Preterm birth | -2.575 | 2.847 | -.061 | .367 | [-9.210; 2.839] |
|  | Child temperament | -0.018 | 0.043 | -.018 | .677 | [-0.106; 0.067] |
|  | COVID-19 pandemic | 0.078 | 0.699 | .004 | .910 | [-1.317; 1.545] |

*Note.* Simple and multiple linear regression analyses based on 5,000 bootstrapping iterations. In Model 2 forced entry was used.

Model 1: adjusted R^2^ = .003, *F*(1,657) = 3.310, *p* = .069; Model 2: adjusted R^2^ = .003, *F*(8,628) = 1.788, *p* = .076

B = unstandardized beta coefficient. SE = standard error for unstandardized beta; ß = standardized beta coefficient;
*p* = bootstrapped p-value; BCa 95% CI = Bias corrected and accelerated 95% confidence interval with α = .05

*^*^*postpartum depression symptoms

*^**^*postpartum anxiety symptoms

**Supplementary Table A.3d**

*Predictive value of mother-child relationship on fine motor development (model 1), and adjusting for seven potential confounding variables (model 2)*

|  | | Fine motor development | | | | |
| --- | --- | --- | --- | --- | --- | --- |
| Model | Predictors | *B* | *SE* | ß | *p* | BCa 95% CI |
| 1 | Mother-child relationship | -0.098 | -0.003 | -.154 | .004 | [-0.168; -0.042] |
| 2 | Mother-child relationship | -0.080 | 0.037 | -.126 | .029 | [-0.158; -0.016] |
|  | Maternal age | -0.077 | 0.077 | -.045 | .310 | [-0.233; 0.079] |
|  | Maternal education | 0.137 | 0.541 | .010 | .801 | [-0.900; 1.278] |
|  | Maternal PPDS*^*^* | -0.089 | 0.092 | -.050 | .342 | [-0.269; 0.086] |
|  | Maternal PPAS*^**^* | -0.116 | 0.116 | -.047 | .322 | [-0.343; 0.119] |
|  | Preterm birth | -2.632 | 1.951 | -.075 | .175 | [-6.797; 1.196] |
|  | Child temperament | -0.018 | 0.034 | -.022 | .588 | [-0.088; 0.053] |
|  | COVID-19 pandemic | 0.354 | 0.600 | .023 | .566 | [-0.805; 1.544] |

*Note.* Simple and multiple linear regression analyses based on 5,000 bootstrapping iterations. In Model 2 forced entry was used.

Model 1: adjusted R^2^ = .022, *F*(1,657) = 16.039, *p* < .001; Model 2: adjusted R^2^ = .030, *F*(8,628) = 3.427, *p* = .001

B = unstandardized beta coefficient. SE = standard error for unstandardized beta; ß = standardized beta coefficient;
*p* = bootstrapped p-value; BCa 95% CI = Bias corrected and accelerated 95% confidence interval with α = .05

*^*^*postpartum depression symptoms

*^**^*postpartum anxiety symptoms

**Supplementary Table A.3e**

*Predictive value of mother-child relationship on problem-solving development (model 1), and adjusting for seven potential confounding variables (model 2)*

|  | | Problem-solving development | | | | |
| --- | --- | --- | --- | --- | --- | --- |
| Model | Predictors | *B* | *SE* | ß | *p* | BCa 95% CI |
| 1 | Mother-child relationship | -0.093 | 0.045 | -.100 | .036 | [-0.185; -0.017] |
| 2 | Mother-child relationship | -0.077 | 0.050 | -.085 | .119 | [-0.185; 0.008] |
|  | Maternal age | -0.056 | 0.100 | -.023 | .580 | [-0.247; 0.142] |
|  | Maternal education | 1.542 | 0.803 | .079 | .056 | [-0.031; 3.158] |
|  | Maternal PPDS*^*^* | -0.032 | 0.125 | -.012 | .801 | [-0.286; 0.208] |
|  | Maternal PPAS*^**^* | -0.282 | 0.176 | -.079 | .109 | [-0.631; 0.082] |
|  | Preterm birth | -1.211 | 2.033 | -.024 | .539 | [-5.541; 3.019] |
|  | Child temperament | -0.041 | 0.050 | -.034 | .423 | [-0.140; 0.053] |
|  | COVID-19 pandemic | 0.801 | 0.881 | .037 | .366 | [-0.919; 2.564] |

*Note.* Simple and multiple linear regression analyses based on 5,000 bootstrapping iterations. In Model 2 forced entry was used.

Model 1: adjusted R^2^ = .009, *F*(1,655) = 6.673, *p* = .010; Model 2: adjusted R^2^ = .009, *F*(8,626) = 2.224, *p* = .024

B = unstandardized beta coefficient. SE = standard error for unstandardized beta; ß = standardized beta coefficient;
*p* = bootstrapped p-value; BCa 95% CI = Bias corrected and accelerated 95% confidence interval with α = .05

*^*^*postpartum depression symptoms

*^**^*postpartum anxiety symptoms

**Supplementary Table A.3f**

*Predictive value of mother-child relationship on personal-social development (model 1), and adjusting for seven potential confounding variables (model 2)*

|  | | Personal-social development | | | | |
| --- | --- | --- | --- | --- | --- | --- |
| Model | Predictors | *B* | *SE* | ß | *p* | BCa 95% CI |
| 1 | Mother-child relationship | -0.051 | 0.034 | -.074 | .131 | [-0.124; 0.008] |
| 2 | Mother-child relationship | -0.067 | -0.001 | -.097 | .073 | [-0.148; 0.002] |
|  | Maternal age | -0.077 | 0.001 | -.041 | .325 | [-0.227; 0.080] |
|  | Maternal education | 0.935 | -0.006 | .063 | .141 | [-0.289; 2.172] |
|  | Maternal PPDS*^*^* | 0.047 | -0.001 | .024 | .623 | [-0.149; 0.231] |
|  | Maternal PPAS*^**^* | 0.215 | 0.005 | .079 | .070 | [-0.037; 0.471] |
|  | Preterm birth | -1.390 | 0.019 | -.037 | .442 | [-5.305; 2.180] |
|  | Child temperament | -0.012 | -0.001 | -.013 | .752 | [-0.081; 0.058] |
|  | COVID-19 pandemic | -0.997 | -0.019 | -.060 | .138 | [-2.257; 0.256] |

*Note.* Simple and multiple linear regression analyses based on 5,000 bootstrapping iterations. In Model 2 forced entry was used.

Model 1: adjusted R^2^ = .004, *F*(1,656) = 3.582, *p* = .059; Model 2: adjusted R^2^ = .008, *F*(8,628) = 1.672, *p* = .102

B = unstandardized beta coefficient. SE = standard error for unstandardized beta; ß = standardized beta coefficient;
*p* = bootstrapped p-value; BCa 95% CI = Bias corrected and accelerated 95% confidence interval with α = .05

*^*^*postpartum depression symptoms

*^**^*postpartum anxiety symptoms

**Supplementary Table A.4a**

*Predictive value of paternal PPOCS^*^ on overall child development (model 1), and adjusting for seven potential confounding variables (model 2)*

|  | | Overall child development | | | | |
| --- | --- | --- | --- | --- | --- | --- |
| Model | Predictors | *B* | *SE* | ß | *p* | BCa 95% CI |
| 1 | Paternal PPOCS | -1.230 | 0.568 | -.143 | .033 | [-2.511; -0.168] |
| 2 | Paternal PPOCS | -1.137 | 0.691 | -.133 | .106 | [-2.561; 0.245] |
|  | Paternal age | -0.343 | 0.379 | -.054 | .376 | [-1.116; 0.429] |
|  | Paternal education | -4.975 | 3.163 | -.078 | .119 | [-11.036; 1.527] |
|  | Paternal PPDS*^**^* | -0.356 | 0.621 | -.036 | .564 | [-1.585; 0.785] |
|  | Paternal PPAS*^***^* | 0.723 | 1.103 | .044 | .503 | [-1.212; 2.914] |
|  | Preterm birth | 0.814 | 6.912 | .005 | .906 | [-12.608; 13.810] |
|  | Child temperament | -0.410 | 0.241 | -.102 | .089 | [-0.854; 0.095] |
|  | COVID-19 pandemic | -1.071 | 3.210 | -.015 | .740 | [-7.476; 5.467] |

*Note.* Simple and multiple linear regression analyses based on 5,000 bootstrapping iterations. In Model 2 forced entry was used.

Model 1: adjusted R^2^ = .018, *F*(1,413) = 8.669, *p* = .003; Model 2: adjusted R^2^ = .024, *F*(8,388) = 2.215, *p* = .026

B = unstandardized beta coefficient. SE = standard error for unstandardized beta; ß = standardized beta coefficient;
*p* = bootstrapped p-value; BCa 95% CI = Bias corrected and accelerated 95% confidence interval with α = .05

*^*^*postpartum obsessive-compulsive symptoms

*^**^*postpartum depression symptoms

*^***^*postpartum anxiety symptoms

**Supplementary Table A.4b**

*Predictive value of paternal PPOCS^*^ on communication development (model 1), and adjusting for seven potential confounding variables (model 2)*

|  | | Communication development | | | | |
| --- | --- | --- | --- | --- | --- | --- |
| Model | Predictors | *B* | *SE* | ß | *p* | BCa 95% CI |
| 1 | Paternal PPOCS | -0.288 | 0.137 | -.122 | .035 | [-0.595; -0.024] |
| 2 | Paternal PPOCS | -0.219 | 0.178 | -.092 | .218 | [-0.592; 0.118] |
|  | Paternal age | 0.048 | 0.097 | .027 | .621 | [-0.155; 0.240] |
|  | Paternal education | 1.089 | 0.925 | .062 | .246 | [-0.645; 2.899] |
|  | Paternal PPDS*^**^* | -0.205 | 0.197 | -.074 | .295 | [-0.630; 0.198] |
|  | Paternal PPAS*^***^* | 0.145 | 0.340 | .032 | .678 | [-0.557; 0.796] |
|  | Preterm birth | 1.259 | 1.396 | .027 | .343 | [-1.851; 3.893] |
|  | Child temperament | 0.013 | 0.073 | .012 | .856 | [-0.132; 0.168] |
|  | COVID-19 pandemic | -1.034 | 0.977 | -.051 | .298 | [-2.779; 0.889] |

*Note.* Simple and multiple linear regression analyses based on 5,000 bootstrapping iterations. In Model 2 forced entry was used.

Model 1: adjusted R^2^ = .013, *F*(1,417) = 6.294, *p* = .012; Model 2: adjusted R^2^ = .006, *F*(8,392) = 1.281, *p* = .252

B = unstandardized beta coefficient. SE = standard error for unstandardized beta; ß = standardized beta coefficient;
*p* = bootstrapped p-value; BCa 95% CI = Bias corrected and accelerated 95% confidence interval with α = .05

*^*^*postpartum obsessive-compulsive symptoms

*^**^*postpartum depression symptoms

*^***^*postpartum anxiety symptoms

**Supplementary Table A.4c**

*Predictive value of paternal PPOCS^*^ on gross motor development (model 1), and adjusting for seven potential confounding variables (model 2)*

|  | | Gross motor development | | | | |
| --- | --- | --- | --- | --- | --- | --- |
| Model | Predictors | *B* | *SE* | ß | *p* | BCa 95% CI |
| 1 | Paternal PPOCS | -0.341 | 0.142 | -.152 | .018 | [-0.646; 0.063] |
| 2 | Paternal PPOCS | -0.262 | 0.194 | -.117 | .179 | [-0.648; 0.114] |
|  | Paternal age | -0.100 | 0.089 | -.060 | .264 | [-0.274; 0.077] |
|  | Paternal education | -1.675 | 0.785 | -.101 | .035 | [-3.223; -0.122] |
|  | Paternal PPDS*^**^* | -0.033 | 0.167 | -.013 | .845 | [-0.366; 0.270] |
|  | Paternal PPAS*^***^* | -0.116 | 0.307 | -.027 | .703 | [-0.714; 0.521] |
|  | Preterm birth | -1.577 | 1.975 | -.036 | .416 | [-5.490; 2.189] |
|  | Child temperament | -0.091 | 0.061 | -.086 | .137 | [-0.199; 0.030] |
|  | COVID-19 pandemic | -0.336 | 0.876 | -.018 | .699 | [-2.059; 1.362] |

*Note.* Simple and multiple linear regression analyses based on 5,000 bootstrapping iterations. In Model 2 forced entry was used.

Model 1: adjusted R^2^ = .021, *F*(1,416) = 9.902, *p* = .002; Model 2: adjusted R^2^ = .031, F(8,391) = 2.572, *p* = .010

B = unstandardized beta coefficient. SE = standard error for unstandardized beta; ß = standardized beta coefficient;
*p* = bootstrapped p-value; BCa 95% CI = Bias corrected and accelerated 95% confidence interval with α = .05

*^*^*postpartum obsessive-compulsive symptoms

*^**^*postpartum depression symptoms

*^***^*postpartum anxiety symptoms

**Supplementary Table A.4d**

*Predictive value of paternal PPOCS^*^ on fine motor development (model 1), and adjusting for seven potential confounding variables (model 2)*

|  | | Fine motor development | | | | |
| --- | --- | --- | --- | --- | --- | --- |
| Model | Predictors | *B* | *SE* | ß | *p* | BCa 95% CI |
| 1 | Paternal PPOCS | -0.117 | 0.093 | -.057 | .209 | [-0.304; 0.064] |
| 2 | Paternal PPOCS | -0.224 | 0.157 | -.110 | .152 | [-0.527; 0.080] |
|  | Paternal age | -0.007 | 0.081 | -.005 | .926 | [-0.168; 0.146] |
|  | Paternal education | -1.199 | 0.742 | -.079 | .109 | [-2.587; 0.290] |
|  | Paternal PPDS*^**^* | 0.189 | 0.149 | .080 | .200 | [-0.102; 0.471] |
|  | Paternal PPAS*^***^* | 0.055 | 0.277 | .014 | .846 | [-0.486; 0.601] |
|  | Preterm birth | 0.962 | 1.728 | .024 | .566 | [-2.484; 4.239] |
|  | Child temperament | -0.045 | 0.057 | -.047 | .430 | [-0.151; 0.066] |
|  | COVID-19 pandemic | 0.816 | 0.855 | .047 | .345 | [-0.828; 2.554] |

*Note.* Simple and multiple linear regression analyses based on 5,000 bootstrapping iterations. In Model 2 forced entry was used.

Model 1: adjusted R^2^ = .001, *F*(1,417) = 1.351, *p* = .246; Model 2: adjusted R^2^ = .000, *F*(8,392) = 0.975, *p* = .455

B = unstandardized beta coefficient. SE = standard error for unstandardized beta; ß = standardized beta coefficient;
*p* = bootstrapped p-value; BCa 95% CI = Bias corrected and accelerated 95% confidence interval with α = .05

*^*^*postpartum obsessive-compulsive symptoms

*^**^*postpartum depression symptoms

*^***^*postpartum anxiety symptoms

**Supplementary Table A.4e**

*Predictive value of paternal PPOCS^*^ on problem-solving development (model 1), and adjusting for seven potential confounding variables (model 2)*

|  | | Problem-solving development | | | | |
| --- | --- | --- | --- | --- | --- | --- |
| Model | Predictors | *B* | *SE* | ß | *p* | BCa 95% CI |
| 1 | Paternal PPOCS | -0.223 | 0.164 | -.076 | .176 | [-0.534; 0.079] |
| 2 | Paternal PPOCS | -0.142 | 0.233 | -.049 | .545 | [-0.596; 0.297] |
|  | Paternal age | -0.161 | 0.124 | -.074 | .193 | [-0.418; 0.080] |
|  | Paternal education | -1.583 | 1.097 | -.073 | .151 | [-3.776; 0.642] |
|  | Paternal PPDS*^**^* | -0.115 | 0.201 | -.034 | .562 | [-0.496; 0.265] |
|  | Paternal PPAS*^***^* | 0.166 | 0.379 | .030 | .655 | [-0.535; 0.967] |
|  | Preterm birth | 2.255 | 2.540 | .038 | .355 | [-2.593; 7.046] |
|  | Child temperament | -0.176 | 0.073 | -.128 | .017 | [-0.315; -0.030] |
|  | COVID-19 pandemic | 0.307 | 1.203 | .012 | .794 | [-2.069; 2.727] |

*Note.* Simple and multiple linear regression analyses based on 5,000 bootstrapping iterations. In Model 2 forced entry was used.

Model 1: adjusted R^2^ = .003, *F*(1,414) = 2.410, *p* = .121; Model 2: adjusted R^2^ = .020, *F*(8,389) = 2.037, *p* = .041

B = unstandardized beta coefficient. SE = standard error for unstandardized beta; ß = standardized beta coefficient;
*p* = bootstrapped p-value; BCa 95% CI = Bias corrected and accelerated 95% confidence interval with α = .05

*^*^*postpartum obsessive-compulsive symptoms

*^**^*postpartum depression symptoms

*^***^*postpartum anxiety symptoms

**Supplementary Table A.4f**

*Predictive value of paternal PPOCS^*^ on personal-social development (model 1), and adjusting for seven potential confounding variables (model 2)*

|  | | Personal-social development | | | | |
| --- | --- | --- | --- | --- | --- | --- |
| Model | Predictors | *B* | *SE* | ß | *p* | BCa 95% CI |
| 1 | Paternal PPOCS | -0.271 | 0.154 | -.118 | .076 | [-0.600; 0.016] |
| 2 | Paternal PPOCS | -0.290 | 0.179 | -.126 | .103 | [-0.649; 0.063] |
|  | Paternal age | -0.099 | 0.097 | -.058 | .313 | [-0.291; 0.099] |
|  | Paternal education | -1.635 | 0.835 | -.096 | .049 | [-3.258; -0.006] |
|  | Paternal PPDS*^**^* | -0.195 | 0.167 | -.073 | .245 | [-0.522; 0.153] |
|  | Paternal PPAS*^***^* | 0.452 | 0.288 | .104 | .109 | [-0.104; 1.007] |
|  | Preterm birth | -0.799 | 2.147 | -.018 | .707 | [-5.572; 3.267] |
|  | Child temperament | -0.117 | 0.058 | -.109 | .041 | [-0.229; 0.009] |
|  | COVID-19 pandemic | -0.882 | 0.881 | -.045 | .320 | [-2.639; 0.854] |

*Note.* Simple and multiple linear regression analyses based on 5,000 bootstrapping iterations. In Model 2 forced entry was used.

Model 1: adjusted R^2^ = .011, *F*(1,417) = 5.841, *p* = .016; Model 2: adjusted R^2^ = .032, *F*(8,392) = 2.628, *p* = .008

B = unstandardized beta coefficient. SE = standard error for unstandardized beta; ß = standardized beta coefficient;
*p* = bootstrapped p-value; BCa 95% CI = Bias corrected and accelerated 95% confidence interval with α = .05

*^*^*postpartum obsessive-compulsive symptoms

*^**^*postpartum depression symptoms

*^***^*postpartum anxiety symptoms

**Supplementary Table A.5**

*Predictive value of paternal PPOCS^*^ on father-child relationship (model 1), and adjusting for potential six confounding variables (model 2)*

|  | | Father-child relationship | | | | |
| --- | --- | --- | --- | --- | --- | --- |
| Model | Predictors | *B* | *SE* | ß | *p* | BCa 95% CI |
| 1 | Paternal PPOCS | 0.746 | 0.134 | .321 | <.001 | [0.483; 1.012] |
| 2 | Paternal PPOCS | 0.207 | -0.001 | .089 | .230 | [-0.143; 0.556] |
|  | Paternal age | -0.148 | 0.001 | -.086 | .051 | [-0.292; 0.000] |
|  | Paternal education | 1.955 | -0.001 | .115 | .009 | [0.580; 3.343] |
|  | Paternal PPDS*^**^* | 0.394 | -0.003 | .147 | .041 | [0.012; 0.761] |
|  | Paternal PPAS*^***^* | 0.596 | 0.022 | .136 | .073 | [-0.056; 1.307] |
|  | Preterm birth | 0.514 | 0.019 | .011 | .708 | [-2.480; 3.416] |
|  | Child temperament | 0.328 | -0.002 | .306 | .000 | [0.231; 0.418] |

*Note.* Simple and multiple linear regression analyses based on 5,000 bootstrapping iterations. In Model 2 forced entry was used.

Model 1: adjusted R^2^ = .101, *F*(1,426) = 48.895, *p* < .001; Model 2: adjusted R^2^ = .249, *F*(7,405) = 20.466, *p* < .001;

B = unstandardized beta coefficient. SE = standard error for unstandardized beta; ß = standardized beta coefficient;
*p* = bootstrapped p-value; BCa 95% CI = Bias corrected and accelerated 95% confidence interval with α = .05

*^*^*postpartum obsessive-compulsive symptoms

*^**^*postpartum depression symptoms

*^***^*postpartum anxiety symptoms

**Supplementary Table A.6a**

*Predictive value of father-child relationship on overall child development (model 1), and adjusting for seven potential confounding variables (model 2)*

|  | | Overall child development | | | | |
| --- | --- | --- | --- | --- | --- | --- |
| Model | Predictors | *B* | *SE* | ß | *p* | BCa 95% CI |
| 1 | Father-child relationship | -0.902 | 0.219 | -.245 | <.001 | [-1.394; -0.450] |
| 2 | Father-child relationship | -0.936 | 0.201 | -.251 | <.001 | [-1.352; -0.540] |
|  | Maternal age | -0.432 | 0.367 | -.067 | .248 | [-1.182; 0.322] |
|  | Paternal education | -2.550 | 3.130 | -.040 | .422 | [-8.712; 3.884] |
|  | Paternal PPDS*^*^* | -0.279 | 0.618 | -.028 | .652 | [-1.557; 0.886] |
|  | Paternal PPAS*^**^* | 0.248 | 0.824 | .015 | .765 | [-1.412; 1.980] |
|  | Preterm birth | 1.627 | 7.159 | .009 | .806 | [-11.968; 15.226] |
|  | Child temperament | -.141 | 0.245 | -.035 | .555 | [-0.587; 0.361] |
|  | COVID-19 pandemic | -1.857 | 3.059 | -.026 | .545 | [-7.929; 4.573] |

*Note.* Simple and multiple linear regression analyses based on 5,000 bootstrapping iterations. In Model 2 forced entry was used.

Model 1: adjusted R^2^ = .058, *F*(1,412) = 26.378, *p* < .001; Model 2: adjusted R^2^ = .063, *F*(8,382) = 4.301, *p* < .001

B = unstandardized beta coefficient. SE = standard error for unstandardized beta; ß = standardized beta coefficient;
*p* = bootstrapped p-value; BCa 95% CI = Bias corrected and accelerated 95% confidence interval with α = .05

*^*^*postpartum depression symptoms

*^**^*postpartum anxiety symptoms

**Supplementary Table A.6b**

*Predictive value of father-child relationship on communication development (model 1), and adjusting for seven potential confounding variables (model 2)*

|  | | Communication development | | | | |
| --- | --- | --- | --- | --- | --- | --- |
| Model | Predictors | *B* | *SE* | ß | *p* | BCa 95% CI |
| 1 | Father-child relationship | -0.123 | 0.069 | -.121 | .075 | [-0.262; 0.018] |
| 2 | Father-child relationship | -0.147 | 0.082 | -.142 | .074 | [-0.323; 0.023] |
|  | Paternal age | 0.028 | 0.102 | .016 | .782 | [-0.183; 0.246] |
|  | Paternal education | 1.498 | 0.938 | .085 | .115 | [-0.313; 3.299] |
|  | Paternal PPDS*^*^* | -0.213 | 0.201 | -.077 | .296 | [-0.618; 0.173] |
|  | Paternal PPAS*^**^* | 0.043 | 0.276 | .009 | .882 | [-0.536; 0.606] |
|  | Preterm birth | 1.468 | 1.449 | .031 | .297 | [-1.667; 4.270] |
|  | Child temperament | 0.057 | 0.076 | .051 | .467 | [-0.092; 0.209] |
|  | COVID-19 pandemic | -1.214 | 0.919 | -.060 | .190 | [-2.981; 0.599] |

*Note.* Simple and multiple linear regression analyses based on 5,000 bootstrapping iterations. In Model 2 forced entry was used.

Model 1: adjusted R^2^ = .012, *F*(1,416) = 6.184, *p* = .013; Model 2: adjusted R^2^ = .017, *F*(8,386) = 1.849, *p* = .067

B = unstandardized beta coefficient. SE = standard error for unstandardized beta; ß = standardized beta coefficient; *p* = bootstrapped p-value; BCa 95% CI = Bias corrected and accelerated 95% confidence interval with α = .05

*^*^*postpartum depression symptoms

*^**^*postpartum anxiety symptoms

**Supplementary Table A.6c**

*Predictive value of father-child relationship on gross motor development (model 1), and adjusting for seven potential confounding variables (model 2)*

|  | | Gross motor development | | | | |
| --- | --- | --- | --- | --- | --- | --- |
| Model | Predictors | *B* | *SE* | ß | *p* | BCa 95% CI |
| 1 | Father-child relationship | -0.250 | 0.058 | -.259 | <.001 | [-0.373; -0.133] |
| 2 | Father-child relationship | -0.251 | 0.063 | -.257 | <.001 | [-0.382; -0.128] |
|  | Paternal age | -0.135 | 0.087 | -.080 | .123 | [-0.308; 0.050] |
|  | Paternal education | -1.080 | 0.793 | -.065 | .172 | [-2.588; 0.450] |
|  | Paternal PPDS*^*^* | -0.006 | 0.161 | -.002 | .967 | [-0.340; 0.308] |
|  | Paternal PPAS*^**^* | -0.192 | 0.228 | -.045 | .392 | [-0.633; 0.289] |
|  | Preterm birth | -1.330 | 1.927 | -.030 | .482 | [-5.053; 2.348] |
|  | Child temperament | -0.011 | 0.065 | -.010 | .869 | [-0.127; 0.115] |
|  | COVID-19 pandemic | -0.568 | 0.844 | -.030 | .493 | [-2.242; 1.187] |

*Note.* Simple and multiple linear regression analyses based on 5,000 bootstrapping iterations. In Model 2 forced entry was used.

Model 1: adjusted R^2^ = .065, *F*(1,415) = 29.943, *p* < .001; Model 2: adjusted R^2^ = .074, *F*(8,385) = 4.904, *p* < .001

B = unstandardized beta coefficient. SE = standard error for unstandardized beta; ß = standardized beta coefficient;
*p* = bootstrapped p-value; BCa 95% CI = Bias corrected and accelerated 95% confidence interval with α = .05

*^*^*postpartum depression symptoms

*^**^*postpartum anxiety symptoms

**Supplementary Table A.6d**

*Predictive value of father-child relationship on fine motor development (model 1), and adjusting for seven potential confounding variables (model 2)*

|  | | Fine motor development | | | | |
| --- | --- | --- | --- | --- | --- | --- |
| Model | Predictors | *B* | *SE* | ß | *p* | BCa 95% CI |
| 1 | Father-child relationship | -0.122 | 0.046 | -.139 | .009 | [-0.212; 0.027] |
| 2 | Father-child relationship | -0.157 | 0.055 | -.178 | .006 | [-0.264; -0.055] |
|  | Paternal age | -0.017 | 0.082 | -.011 | .835 | [-0.192; 0.147] |
|  | Paternal education | -0.706 | 0.743 | -.047 | .345 | [-2.076; 0.752] |
|  | Paternal PPDS*^*^* | 0.196 | 0.150 | .083 | .191 | [-0.115; 0.485] |
|  | Paternal PPAS*^**^* | -0.058 | 0.210 | -.015 | .780 | [-0.471; 0.357] |
|  | Preterm birth | 1.127 | 1.739 | .028 | .504 | [-2.348; 4.517] |
|  | Child temperament | -0.003 | 0.059 | -.003 | .964 | [-0.114; 0.123] |
|  | COVID-19 pandemic | 0.687 | 0.839 | .040 | .419 | [-1.041; 2.441] |

*Note.* Simple and multiple linear regression analyses based on 5,000 bootstrapping iterations. In Model 2 forced entry was used.

Model 1: adjusted R^2^ = .017, *F*(1,416) = 8.244, *p* = .004; Model 2: adjusted R^2^ = .017, *F*(8,386) = 1.864, *p* = .064

B = unstandardized beta coefficient. SE = standard error for unstandardized beta; ß = standardized beta coefficient;
*p* = bootstrapped p-value; BCa 95% CI = Bias corrected and accelerated 95% confidence interval with α = .05

*^*^*postpartum depression symptoms

*^**^*postpartum anxiety symptoms

**Supplementary Table A.6e**

*Predictive value of father-child relationship on problem-solving development (model 1), and adjusting for seven potential confounding variables (model 2)*

|  | | Problem-solving development | | | | |
| --- | --- | --- | --- | --- | --- | --- |
| Model | Predictors | *B* | *SE* | ß | *p* | BCa 95% CI |
| 1 | Father-child relationship | -0.271 | 0.063 | -.218 | <.001 | [-0.396; -0.151] |
| 2 | Father-child relationship | -0.265 | 0.075 | -.211 | .001 | [-0.411; -0.121] |
|  | Paternal age | -0.180 | 0.123 | -.083 | .152 | [-0.424; 0.048] |
|  | Paternal education | -1.080 | 1.085 | -.051 | .318 | [-3.199; 1.170] |
|  | Paternal PPDS*^*^* | -0.026 | 0.193 | -.008 | .895 | [-0.407; 0.354] |
|  | Paternal PPAS*^**^* | 0.187 | 0.290 | .034 | .511 | [-0.373; 0.831] |
|  | Preterm birth | 2.242 | 2.600 | .038 | .367 | [-2.697; 7.093] |
|  | Child temperament | -0.095 | 0.076 | -.071 | .207 | [-0.243; 0.052] |
|  | COVID-19 pandemic | 0.260 | 1.189 | .011 | .816 | [-2.086; 2.766] |

*Note.* Simple and multiple linear regression analyses based on 5,000 bootstrapping iterations. In Model 2 forced entry was used.

Model 1: adjusted R^2^= .045, *F*(1,413) = 20.513, *p* < .001; Model 2: adjusted R^2^ = .054, *F*(8,383) = 3.799, *p* < .001

B = unstandardized beta coefficient. SE = standard error for unstandardized beta; ß = standardized beta coefficient;
*p* = bootstrapped p-value; BCa 95% CI = Bias corrected and accelerated 95% confidence interval with α = .05

*^*^*postpartum depression symptoms

*^**^*postpartum anxiety symptoms

**Supplementary Table A.6f**

*Predictive value of father-child relationship on personal-social development (model 1), and adjusting for seven potential confounding variables (model 2)*

|  | | Personal-social development | | | | |
| --- | --- | --- | --- | --- | --- | --- |
| Model | Predictors | *B* | *SE* | ß | *p* | BCa 95% CI |
| 1 | Father-child relationship | -0.147 | 0.055 | -.150 | .010 | [-.266; -.041] |
| 2 | Father-child relationship | -0.124 | 0.054 | -.125 | .020 | [-0.237; -0.019] |
|  | Paternal age | -0.107 | 0.098 | -.063 | .267 | [-0.304; 0.093] |
|  | Paternal education | -1.116 | 0.840 | -.066 | .180 | [-2.807; 0.664] |
|  | Paternal PPDS*^*^* | -0.229 | 0.161 | -.087 | .151 | [-0.560; 0.082] |
|  | Paternal PPAS*^**^* | 0.257 | 0.241 | .059 | .264 | [-0.240; 0.726] |
|  | Preterm birth | -0.583 | 2.175 | -.013 | .787 | [-5.206; 3.590] |
|  | Child temperament | -0.091 | 0.060 | -.085 | .133 | [-0.205; 0.028] |
|  | COVID-19 pandemic | -1.018 | 0.902 | -.053 | .262 | [-2.805; 0.868] |

*Note.* Simple and multiple linear regression analyses based on 5,000 bootstrapping iterations. In Model 2 forced entry was used.

Model 1: adjusted R^2^ = .020, *F*(1,416) = 9.519, *p* = .002; Model 2: adjusted R^2^ = .036, *F*(8,386) = 2.827, *p* = .005

B = unstandardized beta coefficient. SE = standard error for unstandardized beta; ß = standardized beta coefficient;
*p* = bootstrapped p-value; BCa 95% CI = Bias corrected and accelerated 95% confidence interval with α = .05

*^*^*postpartum depression symptoms

*^**^*postpartum anxiety symptoms
