# Supplementary material for: The impact of postpartum obsessive-compulsive symptoms on child development and the mediating role of the parent–child relationship: A prospective longitudinal study
Source: Front Psychiatry. 2022 Aug 16;13:886347. doi: 10.3389/fpsyt.2022.886347 (PMC9532008; doi:10.3389/fpsyt.2022.886347)
Supplement: Supplementary file 2 [file Table_2.DOCX]

**Supplementary Material B: Full results of the mediation analyses**

**Supplementary Table B.1a**

*The mediating role of the mother-child relationship (M) between maternal PPOCS^*^ (X) and child development (Y)*

|  | Total effect of X on Y (c) | | | | Direct effect of X on Y (c’) | | | | Indirect effect of X on Y (ab) | | |
| --- | --- | --- | --- | --- | --- | --- | --- | --- | --- | --- | --- |
| Outcome variable (Y) | B | SE | *p* | 95% CI | B | SE | *p* | 95% CI | B | SE | 95% CI |
| Overall development | -0.647 | 0.256 | .012 | [-1.150; -0.144] | -0.441 | 0.265 | .097 | [-0.961; -0.079] | -0.206 | 0.100 | [-0.424; -0.026] |
| Communication development | -0.141 | 0.088 | .108 | [-0.314; 0.031] | -0.116 | 0.092 | .205 | [-0.296; 0.064] | -0.025 | 0.028 | [-0.084; 0.028] |
| Gross motor development | -0106 | 0.073 | .165 | [-0.256; 0.044] | -0.077 | 0.079 | .336 | [-0.232; 0.079] | -0.030 | 0.023 | [-0.079; 0.012] |
| Fine motor development | -0.166 | 0.063 | .009 | [-0.289; -0.042] | -0.105 | 0.065 | .108 | [-0.232; 0.023] | -0.061 | -0.024 | [-0.112; -0.019] |
| Problem-solving development | -0.238 | 0.091 | .009 | [-0.417; -0.059] | -0.185 | 0.095 | .051 | [-0.371; 0.001] | -0.053 | 0.030 | [-0.116; 0.005] |
| Personal-social development | -0.003 | 0.069 | .971 | [-0.133; 0.138] | 0.040 | 0.072 | .577 | [-0.101; 0.181] | -0.038 | 0.025 | [-0.091; 0.007] |

*Note.* Simple mediation analyses using PROCESS model 4 based on 5,000 bootstrapping iterations; B = unstandardized beta coefficient. SE = standard error for unstandardized beta; *p* = p-value; 95% CI = 95% percentile confidence interval with α = .05

Effects in bold are statistically significant (i.e., *p* < .05 or bootstrapped 95% CI not crossing 0)

*^*^*postpartum obsessive-compulsive symptoms

**Supplementary Table B.1b**

*The mediating role of the mother-child relationship (M) between maternal PPOCS^*^ (X) and child development (Y), adjusting for maternal age, education, PPDS^**^, and PPAS^***^, preterm birth, child temperament, and COVID-19 pandemic-driven adversities*

|  | Total effect of X on Y (c) | | | | Direct effect of X on Y (c’) | | | | Indirect effect of X on Y (ab) | | | |
| --- | --- | --- | --- | --- | --- | --- | --- | --- | --- | --- | --- | --- |
| Outcome variable (Y) | B | SE | *p* | 95% CI | B | SE | *p* | 95% CI | B | SE | 95% CI | |
| Overall development | -0.370 | 0.342 | .280 | [-1.042; 0.302] | -0.291 | 0.343 | .397 | [-0.965; 0.383] | -0.079 | 0.055 | [-0.195; 0.024] |  |
| Communication development | -0.112 | 0.118 | .344 | [-0.343; 0.120] | -0.104 | 0.119 | .382 | [-0.337; 0.129] | -0.008 | 0.015 | [-0.034; 0.024] | |
| Gross motor development | 0.049 | 0.102 | .631 | [-0.151; 0.249] | 0.055 | 0.103 | .594 | [-0.147; 0.256] | -0.006 | 0.012 | [-0.031; 0.019] | |
| Fine motor development | -0.073 | 0.084 | .387 | [-0.237; 0.092] | -0.049 | 0.084 | .562 | [-0.214; 0.116] | -0.024 | 0.014 | [-0.054; 0.002] | |
| Problem-solving development | -0.125 | 0.121 | .302 | [-0.362; 0.112] | -0.103 | 0.121 | .394 | [-0.341; 0.135] | -0.021 | 0.007 | [-0.025; 0.004] | |
| Personal-social development | -0.109 | 0.091 | .234 | [-0.288; 0.071] | -0.088 | 0.092 | .336 | [-0.268; 0.092 | -0.021 | 0.014 | [-0.050; 0.005] | |

*Note.* Simple mediation analyses using PROCESS model 4 based on 5,000 bootstrapping iterations; B = unstandardized beta coefficient. SE = standard error for unstandardized beta; *p* = p-value; 95% CI = 95% percentile confidence interval with α = .05

*^*^*postpartum obsessive-compulsive symptoms

*^**^*postpartum depression symptoms

*^***^*postpartum anxiety symptoms

**Supplementary Table B.2a**

*The mediating role of the father-child relationship (M) between paternal PPOCS^*^ (X) and child development (Y)*

|  | Total effect of X on Y (c) | | | | Direct effect of X on Y (c’) | | | | Indirect effect of X on Y (ab) | | |
| --- | --- | --- | --- | --- | --- | --- | --- | --- | --- | --- | --- |
| Outcome variable (Y) | B | SE | *p* | 95% CI | B | SE | *p* | 95% CI | B | SE | 95% CI |
| Overall development | -1.258 | 0.417 | .003 | [-2.076; -0.439] | -0.643 | 0.430 | .136 | [-1.488; 0.203] | -0.615 | 0.196 | [-1.041; -0.283] |
| Communication development | -0.290 | -0.116 | .013 | [-0.517; -0.062] | -0.221 | 0.122 | .070 | [-0.461; 0.018] | -0.068 | 0.055 | [-0.187; 0.031] |
| Gross motor development | -0.345 | 0.109 | .002 | [-0.559; -0.122] | -0.178 | 0.112 | .113 | [-0.398; 0.042] | -0.167 | 0.053 | [-0.281; -0.075] |
| Fine motor development | -0.119 | 0.101 | .237 | [-0.317; 0.079] | -0.027 | 0.105 | .801 | [-0.234; 0.181] | -0.093 | 0.043 | [-0187; -0.019] |
| Problem-solving development | -0.234 | 0.142 | .101 | [-0.513; 0.046] | -0.031 | 0.147 | .831 | [-0.320; 0.258] | -0.203 | 0.064 | [-0.342; -0.091] |
| Personal-social development | -0.279 | 0.112 | .013 | [-0.499; -0.059] | -0.183 | 0.117 | .120 | [-0.414; 0.048] | -0.096 | 0.054 | [-0.199; -0.019] |

*Note.* Simple mediation analyses using PROCESS model 4 based on 5,000 bootstrapping iterations; B = unstandardized beta coefficient. SE = standard error for unstandardized beta; *p* = p-value; 95% CI = 95% percentile confidence interval with α = .05

Effects in bold are statistically significant (i.e., *p* < .05 or bootstrapped 95% CI not crossing 0)

*^*^*postpartum obsessive-compulsive symptoms

**Supplementary Table B.2b**

*The mediating role of the father-child relationship (M) between paternal PPOCS^*^ (X) and child development (Y), adjusting for paternal age, education, PPDS^**^, and PPAS^***^, preterm birth, child temperament, and COVID-19 pandemic-driven adversities*

|  | Total effect of X on Y (c) | | | | Direct effect of X on Y (c’) | | | | Indirect effect of X on Y (ab) | | |
| --- | --- | --- | --- | --- | --- | --- | --- | --- | --- | --- | --- |
| Outcome variable (Y) | B | SE | *p* | 95% CI | B | SE | *p* | 95% CI | B | SE | 95% CI |
| Overall development | -1.206 | 0.655 | .066 | [-2.493; 0.082] | -1.009 | 0.641 | .116 | [-2.269; 0.252] | -0.197 | 0.178 | [-0.579; 0.121] |
| Communication development | -0.240 | 0.186 | .197 | [-0.605; 0.125] | -0.208 | 0.185 | .262 | [-0.572; 0.156] | -0.0321 | 0.037 | [-0.124; 0.017] |
| Gross motor development | -0.265 | 0.174 | .129 | [-0.607; 0.077] | -0.210 | 0.170 | .218 | [-0.544; 0.125] | -0.055 | 0.047 | [-0.157; 0.035] |
| Fine motor development | -0.234 | 0.160 | .146 | [-0.549; 0.082] | -0.199 | 0.159 | .211 | [-0.512; 0.114] | -0.034 | 0.032 | [-0.107; 0.017] |
| Problem-solving development | -0.143 | 0.219 | .515 | [-0.573; 0.288] | -0.084 | 0.216 | .698 | [-0.508; 0.340] | -0.059 | 0.054 | [-0.180; 0.033] |
| Personal-social development | -0.333 | 0.175 | .057 | [-0.676; 0.010] | -0.307 | 0.174 | .079 | [-0.650; 0.035] | -0.026 | 0.027 | [-0.090; 0.014] |

*Note.* Simple mediation analyses using PROCESS model 4 based on 5,000 bootstrapping iterations; B = unstandardized beta coefficient. SE = standard error for unstandardized beta; *p* = p-value; 95% CI = 95% percentile confidence interval with α = .05

*^*^*postpartum obsessive-compulsive symptoms

*^**^*postpartum depression symptoms

*^***^*postpartum anxiety symptoms
